# Dendritic Membranized Coacervate Microdroplets: A Robust Platform for Synthetic-Living Cell Consortia

**DOI:** 10.1021/jacs.5c09772

**Published:** 2025-08-02

**Authors:** Celia Jimenez-Lopez, Lucas Garcia-Abuin, Eduardo Fernandez-Megia

**Affiliations:** Centro Singular de Investigación en Química Biolóxica e Materiais Moleculares (CIQUS), Departamento de Química Orgánica, 16780 Universidade de Santiago de Compostela, Jenaro de la Fuente s/n, Santiago de Compostela 15782, Spain

## Abstract

Bottom-up synthetic biology seeks to construct artificial cells with biomimetic or novel functionalities to uncover the fundamental principles of cellular evolution and drive advances in medicine and bioengineering. Among them, membranized coacervate microdroplets (MCM) uniquely combine a molecularly crowded aqueous interior with a surrounding membrane, both hallmarks of eukaryotic cells. Replicating cellular functions requires synthetic cells to remain structurally stable in biological environments, where ionic strength presents a significant threat to the integrity of complex coacervates. By leveraging the globular and rigid architecture of dendrimers, MCM, composed of oppositely charged small dendrimers and polypeptides—further stabilized by a charged PEG-dendritic copolymer assembled at the periphery—exhibits a critical salt concentration more than twice that of coacervates formed from polypeptides or branched polyelectrolytes with significantly higher degrees of polymerization. This highlights the enhanced robustness of dendritic MCM under physiological conditions and their suitability as synthetic cells in biological media. By mimicking key cell-like behavior such as efficient enzyme encapsulation (irrespective of the isoelectric point), fast internal dynamics, and chemical communication, dendritic MCM emerge as a promising synthetic cell platform for the selective delivery of therapeutic enzymes. In addition, their ability to engage in signal transduction pathways within synthetic-natural cell consortia, enabling responses to extracellular cues via chemical signaling, paves their way in tissue engineering and regenerative medicine.

## Graphical abstract

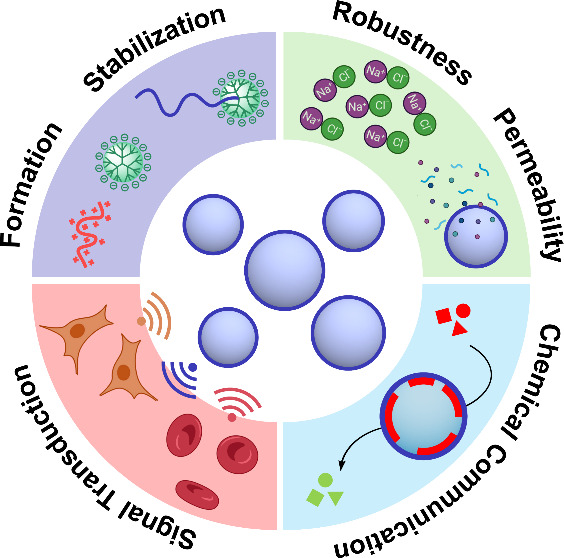

## Introduction

Bottom-up synthetic biology^1^ aims to engineer artificial cell models from synthetic and natural components, incorporating biomimetic or entirely novel functionalities.^2–4^ Reducing the complexity of natural cells offers promising avenues for uncovering the fundamental principles underlying cellular evolution and driving advances in medicine and bioengineering.^5–7^ Despite the extensive reliance of synthetic cells on vesicles for their ability to mimic natural membranes, they fall short in replicating the molecularly crowded interior that characterizes eukaryotic cells.^8^ Complex coacervates, originally proposed by Oparin,^9,10^ represent an intriguing alternative. Coacervates are microdroplets formed spontaneously by phase separation of oppositely charged polyelectrolytes in water.^11,12^ They have a highly concentrated aqueous interior, enriched with biomolecules and small molecules that closely mimics the intracellular milieu, surrounded by a diluted environment.^13,14^ The structural versatility of coacervates makes them attractive models for synthetic cells with multiple biomimetic functions.^7,15^ However, a key challenge with coacervates is their inherent lack of an enclosing membrane. In the absence of interfacial stabilization, coacervates tend to coalesce, restricting their applicability. Pioneering work by the groups of Mann, Keating, and van Hest has overcome this limitation by developing membrane-stabilized coacervates with auxiliary fatty acids,^16^ phospholipids,^17^ polymers,^18^ liposomes,^19^ inorganic nanoparticles,^20^ proteins,^21^ or even living bacteria^22^ on the surface that prevent aggregation while preserving permeability to small molecules. The resulting membranized coacervate microdroplets (MCM) prove to be hybrid systems, combining the advantages of vesicle- and coacervate-based models in a single construct.^23^

To replicate cellular functions, synthetic cells must retain their structure and properties in biological media.^3^ Modeling cell-like behavior, such as compartmentalization, energy supply and metabolism, gene replication, biosynthesis, communication, growth and division or motility, requires stability in the extracellular environment.^6^ For coacervates, this is especially dependent on their resistance to variations in ionic strength. While low concentrations of salts favor coacervation, higher concentrations destabilize phase separation.^24,25^ Ironically, the driving force behind the formation of complex coacervates is also a threat to their stability. With the aim of increasing coacervate resistance to salt, we focus on dendrimers: tree-like polymers with a globular architecture, synthesized with unprecedented control over size and multivalency.^26,27^ Recently, charged dendrimers have been described to increase the stability of nanosized, coacervate-like polyion complex (PIC) micelles.^28–30^ Like coacervates, PIC micelles suffer from low salt stability, often disassembling at 150 mM NaCl, characteristic of physiological conditions.^31^ However, when charged dendrimers are incorporated into PIC micelles, unprecedented stability in serum and toward ionic strength has been reported.^32–39^ Fundamental differences in local dynamics between linear polymers and dendrimers^40^ explain this “dendritic effect”.^41^ Whereas the local dynamics of linear polymers are governed by repeating segments and remain independent of molecular weight, dendrimer dynamics depend on the dendritic generation, and hence molecular weight.^40^ Therefore, incorporating charged dendrimers into coacervates offers a promising strategy for enhancing their stability under physiological conditions. In addition, although the monodispersity of dendrimers makes them ideal candidates for evaluating new technologies and bioapplications,^42^ their use in the construction of coacervate-based synthetic cells has remained surprisingly unexplored. This is particularly noteworthy because dendrimers are regarded as mimetics of proteins,^41^ which make up about 30% mass of the cytoplasm.^43^

Herein, we describe robust dendritic MCM formed through a two-stage coacervation process shown in Figure 1: an initial complexation of 3­[G2]-Bz – a dendrimer of the gallic acid-triethylene glycol (GATG)^36,44^ family with 27 peripheral carboxylates – and poly-l-lysine (PLL, DP 101), followed by an interfacial stabilization of the resulting coacervates with PEG­[G3]-Bz – a PEG_5k_-dendritic block copolymer with 27 carboxylates (PEG is poly­(ethylene glycol)). This strategy endows dendritic MCM with superior salt resistance compared to coacervates formed from linear polyamines and polycarboxylates with an even higher multivalency. The ability of dendritic MCM to emulate cell-like behaviors, such as compartmentalization, enzyme encapsulation, and biochemical reactions, positions them as synthetic cell models capable of chemical communication with neighboring living cells.

**Figure 1:**
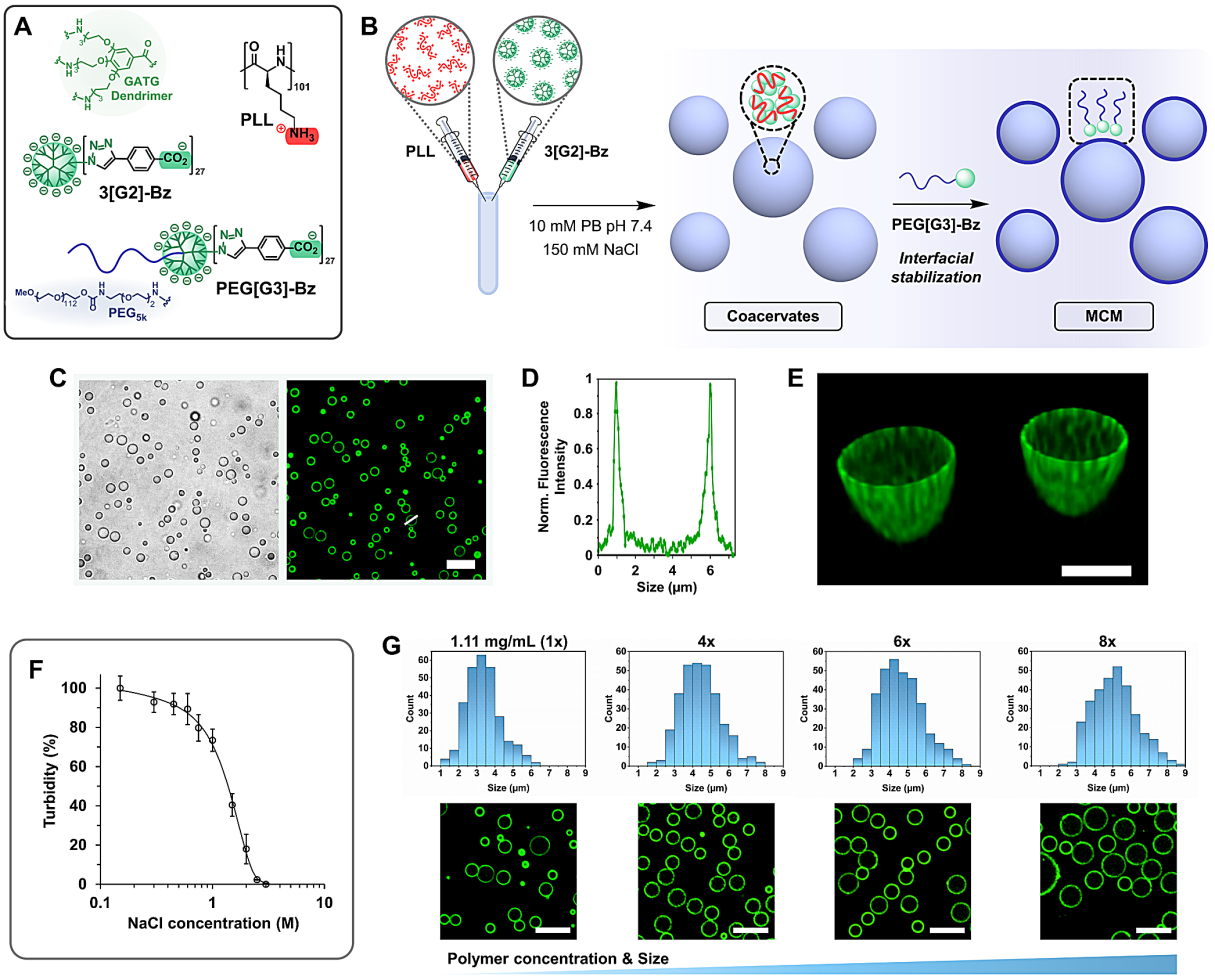
Structure of 3­[G2]-Bz, PEG­[G3]-Bz, and PLL (A). Schematic representation of the complex coacervation of 3­[G2]-Bz and PLL and subsequent interfacial stabilization with PEG­[G3]-Bz (B). Brightfield and confocal laser scanning microscopy (CLSM) images of dendritic membranized coacervate microdroplets (MCM). CLSM image shows AF488-PEG­[G3]-Bz (green) hierarchically assembled at the external interface of the droplets. Scale bar 10 μm (C). Cross sectional AF488 fluorescence intensity profile (line in C) (D). 3D reconstructed image showing selective peripheral localization of AF488-PEG­[G3]-Bz. Scale bar 2.5 μm (E). Ionic strength stability of dendritic MCM analyzed by turbidity measurements (F). Size distributions and CLSM images of MCM prepared at increasing polymer concentrations. MCM interfacially stabilized with AF488-PEG­[G3]-Bz (green). Scale bars 10 μm (G).

## Results and Discussion

### Preparation and Interfacial Stabilization of Dendritic Coacervates

Dendritic coacervates were prepared by mixing solutions of 3­[G2]-Bz and PLL at a stoichiometric charge ratio of carboxylate and ammonium groups, in 10 mM phosphate buffer (PB) pH 7.4, 150 mM NaCl (Figures 1A and 1B). Although the interaction between the oppositely charged polyelectrolytes resulted in a phase separation, the lifetime of this turbid coacervate suspension was short, with coacervates coalescing into a bulk phase after only 2 h. Conversely, long-term stability was achieved via interfacial stabilization with PEG­[G3]-Bz added to the suspension 40 min after mixing (Figure 1B). The copolymer hierarchically assembled on the surface of the coacervate microdroplets leads to a stable dispersion of PEGylated MCM with a mean diameter of 3.60 ± 1.02 μm as determined by optical microscopy (Figure 1C). The effectiveness of the stabilization was immediately apparent as the lifetime of the droplets increased from minutes to several days (Figure S1A). Coacervate membranization was confirmed by confocal laser scanning microscopy (CLSM) using AF488-PEG­[G3]-Bz, a fluorescently labeled version of the block copolymer incorporating Alexa Fluor 488 (AF488, green) at the distal end of the PEG block. Figures 1C-1E show a well-defined MCM organization with a continuous green coating membrane, indicating that PEG chains from PEG­[G3]-Bz are selectively exposed at the external interface of the coacervates, while 3­[G2]-Bz and PLL are confined within the droplets.

The coacervate aging time prior to the addition of PEG­[G3]-Bz and the amount of copolymer used were both optimized. A 40 min coacervation time was selected, as shorter or longer durations resulted in smaller MCM with high tendency to aggregate and coalesce after 24 h. This behavior is consistent with previous findings on the interfacial stabilization of MCM,^18^ as well as with reports from our group and others^39,45^ describing the formation of PIC micelles as a two-step process, involving an initial kinetic step followed by a second equilibration process. In our case, the aggregation of 3­[G2]-Bz and PLL to form minimum polyelectrolyte complexes, which subsequently assemble to induce phase separation. An amount of PEG­[G3]-Bz equivalent to 9 mol % of 3­[G2]-Bz was determined as optimal for coacervate stabilization. Although lower concentrations of PEG­[G3]-Bz had no immediate effect on the size and number of prepared droplets, a reduction in long-term turbidity was seen, indicative of a less efficient stabilization (Figure S1B). Interestingly, coacervates were also found to be efficiently stabilized by a cationic PEG-dendritic block copolymer as AF488-PEG­[G3]-NH_2_·HCl, functionalized with 27 peripheral ammonium groups (Figure S2), indicating the generalizability of the strategy independently of the copolymer charge.

Complex coacervation is strongly dependent on the ionic strength of the medium. A critical salt concentration is defined as the threshold above which no phase separation is observed.^24,25^ To assess the ionic strength stability conferred by the rigid dendritic architecture, MCM were treated with increasing concentrations of NaCl and the effect was analyzed by turbidity. As shown in Figure 1F, the addition of salt leads to a slight decrease in turbidity up to 0.6 M, followed by a more pronounced decay until a critical NaCl concentration is reached above 2.5 M. This concentration is more than twice that of coacervates prepared from PLL – or branched poly­(ethylenimine) – and anionic polypeptides (poly-l-glutamic and poly-l-aspartic acid) with significantly higher degrees of polymerization,^46–49^ which highlights the robustness of dendritic MCM under physiological conditions and their potential as a synthetic cell platform in biological media. Complex coacervation is also influenced by polymer concentration, with higher concentrations leading to larger droplet sizes,^46,47^ a property exploited to tune the size of dendritic MCM. Droplets prepared with polymer concentrations ranging from 1.1 to ca. 9.0 mg/mL – maintaining a stoichiometric charge ratio – were analyzed by confocal and brightfield microscopy, which showed an increase in size from 3.60 ± 1.02 to 5.25 ± 1.16 μm while preserving a spherical morphology and selective membranization (Figures 1G and S3).

### Cytomimetic Functions of Dendritic MCM: Protein Encapsulation and Internal Dynamics

A fundamental property of complex coacervates is their ability to efficiently partition biomacromolecules, such as proteins and nucleic acids, into the polymeric rich phase. This internal organization, which mimics the compartmentalization of living cells, creates specific microenvironments to regulate biochemical reactions. The potential of dendritic MCM to emulate life-like technologies, such as enzyme encapsulation and chemical communication, was assessed using a well-established signal transduction pathway: the enzymatic cascade reaction composed of glucose oxidase (GOX, 160 kDa, pI 4.2; pI is the isoelectric point) and horseradish peroxidase (HRP, 44 kDa, pI 9.0). Both enzymes fluorescently labeled with Cyanine 5 (GOX-Cy5 and HRP-Cy5) were encapsulated into MCM by addition to the coacervate mixture immediately after the polyelectrolytes. Following interfacial stabilization, GOX-Cy5@MCM and HRP-Cy5@MCM were obtained with very high encapsulation efficiencies (EE, 82% for GOX and 76% for HRP), regardless of the protein molecular weight and pI – the latter facilitated by the stoichiometric charge ratio used during coacervation. CLSM experiments showed a selective localization of the enzymes, primarily driven by the protein charge (Figure 2, Cy5 is shown red). While HRP-Cy5 predominantly distributes within the crowded coacervate interior (Figure 2A), GOX-Cy5 locates at the MCM periphery forming discrete patches (Figure 2C). This latter arrangement, also observed for other negatively charged proteins, such as bovine serum albumin (BSA, 66 kDa, pI 4.7; EE 82%) and insulin (6 kDa, pI 5.3; EE 71%) (Figures 2D and 2E), is consistent with the formation of a second coacervate phase where negatively charged proteins are largely found, independently of their molecular weight. Similarly to HRP, lysozyme (14 kDa, pI 11.4; EE 81%), another positively charged protein, also localizes homogeneously throughout the coacervate (Figure 2B). Competition between GOX and the other negatively charged proteins with 3­[G2]-Bz for binding to PLL results in a patchy phase, separated from the main coacervate phase. Although similar multiphase patterns have been described for coacervates of PLL and oligonucleotides (RNA and DNA),^49,50^ to the best of our knowledge, such distributions have not previously been observed for proteins. While these experiments need to be extended to other proteins, we believe that the rigid and globular structure of the dendrimer precisely regulates this spatial arrangement, which could be particularly useful for studying protein functions associated with cell membranes. Notably, coencapsulation of GOX and HRP in a single coacervate retained their selective localization with comparable EE: 76% for GOX-Cy5 (red) and 67% for HRP-RITC (functionalized with rhodamine B isothiocyanate, cyan) (Figure 2F).

**Figure 2:**
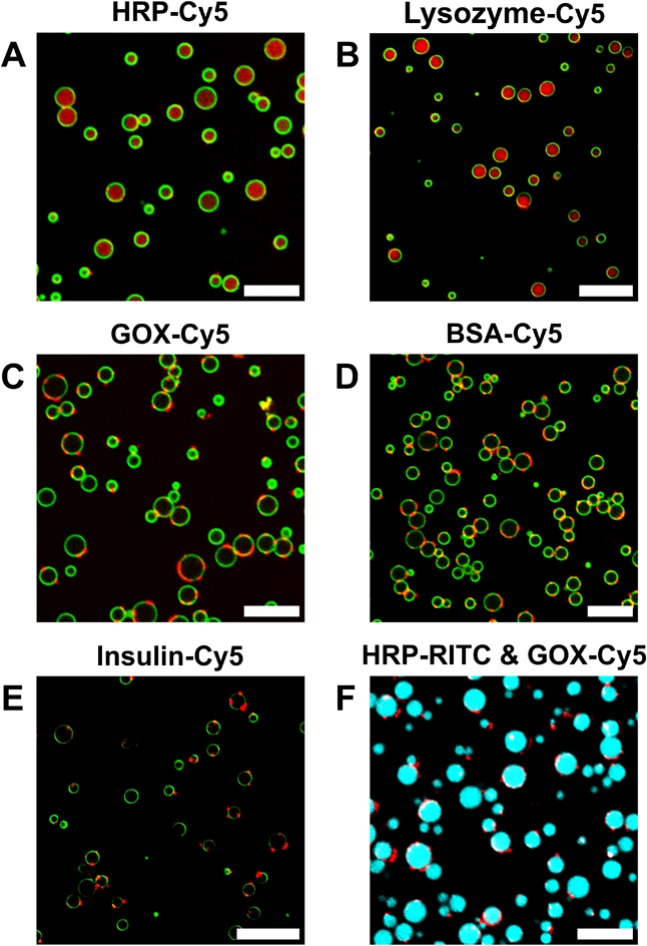
CLSM images of fluorescently labeled enzyme-loaded MCM show selective enzyme localization: HRP (A, F) and lysozyme (B) distributed within the coacervate and GOX (C, F), BSA (D), and insulin (E) forming discrete localized patches at the MCM periphery. Scale bars 10 μm, AF488-PEG­[G3]-Bz (green), Cy5 (red), RITC (cyan). Images of individual channels are shown in Figure S4.

The ability of distinct dendritic MCM populations to coexist was then verified. Fluorescently labeled MCM were prepared using/encapsulating PLL-Cy5 (red), PLL-AF488 (green), HRP-Cy5 (red), GOX-Cy5 (red), and HRP-RITC (cyan), and the transfer of macromolecular components between populations was analyzed by time-dependent CLSM. Interestingly, while migration of polyelectrolytes and loaded proteins was not observed between single-phase MCM (PLL-Cy5 versus PLL-AF488 in Figure 3A, HRP-Cy5 versus PLL-AF488 in Figure 3B), transfer of macromolecules from single-phase (PLL-AF488 and HRP-RITC) toward GOX-loaded multiphase droplets was seen (Figures 3C and 3D). These results underscore the ability of dendritic MCM to compartmentalize biomacromolecules, mimicking the complex and dynamic cellular scenario. Regulation of migration between coacervates in response to a protein-rich phase provides an interesting model for studying partitioning among microenvironments, exploiting the known ability of dendrimers to selectively bind proteins.^51,52^

**Figure 3:**
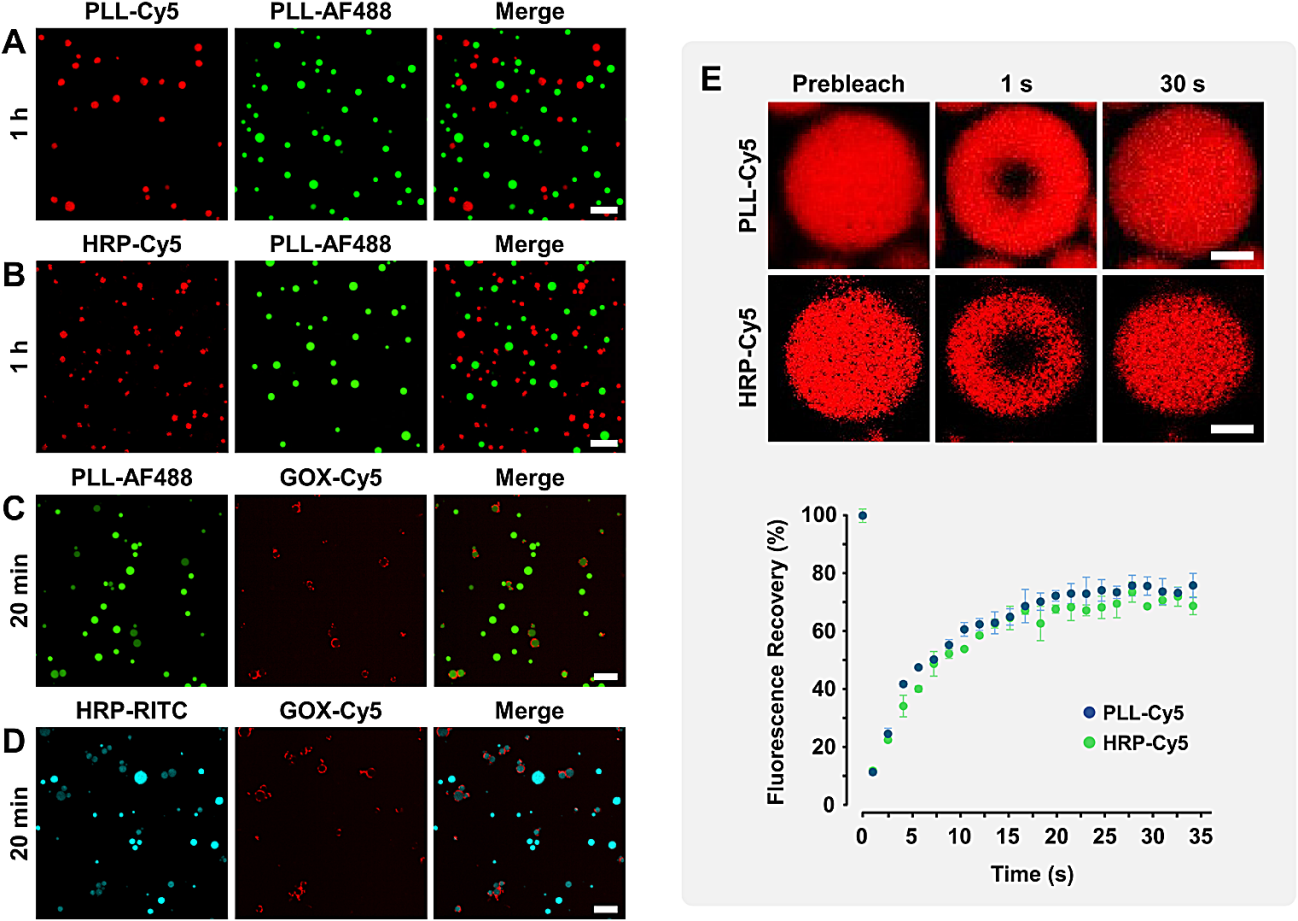
CLSM images of pairs of fluorescently labeled MCM populations prepared/loaded with PLL-Cy5 (red), PLL-AF488 (green), HRP-Cy5 (red), GOX-Cy5 (red), and HRP-RITC (cyan) taken at varying time points after mixing. Scale bars 10 μm (A–D). Images of fluorescence recovery after photobleaching (FRAP) experiments of PLL-Cy5/MCM and HRP-Cy5@MCM and fluorescence recovery plots. Scale bars 1.5 μm (E).

Phase fluidity is a key consideration in designing coacervates that mimic natural cell behavior. Maintaining enzyme activity upon encapsulation requires effective diffusion within the spatially confined coacervate core. The internal mobility of macromolecular species within dendritic MCM was studied by fluorescence recovery after photobleaching (FRAP) experiments (Figure 3E). In a FRAP experiment, fluorescent molecules within a region of interest are irreversibly photobleached by transient exposure to a laser beam. The analysis of the fluorescence recovery within that region due to the internal mobility of the molecules provides information about their diffusion rate (see the SI).^53^ Independent populations of PLL-Cy5/MCM and HRP-Cy5@MCM were selected to compare the mobility of a polyelectrolyte scaffold and a model encapsulated protein cargo. Figure 3E shows fast fluorescence recoveries for both species. Fitting the FRAP recovery curves to an exponential function (Figure S5) yielded very similar fluorescence recovery half-times (*t*_1/2_) of 4.55 ± 0.25 and 4.72 ± 0.24 s for PLL and HRP, respectively. These values reflect a liquid-like state inside the droplets despite the rigid 3­[G2]-Bz architecture. Determination of the apparent diffusion coefficients (*D*_app_) afforded an identical value of 0.017 ± 0.001 μm^2^/s for PLL and HRP, independently of their MW, suggesting compensating effects of conformation and net charge. This value is two to 3 orders of magnitude lower than the *D*_app_ determined by FRAP for proteins in the cytoplasm and nuclei of various cell lines.^54–58^ Given the known relationship between cell activity and viscosity,^59^ future studies will focus on modulating macromolecular diffusion within MCM by tuning the molecular weight of PLL and the dendritic generation.^60,61^

### Cytomimetic Functions of Dendritic MCM: Chemical Communication

Having demonstrated that dendritic MCM efficiently encapsulate biomacromolecules, their permeability to small molecules and ability to process chemical signals was assessed using the GOX-HRP enzymatic cascade (Figure 4A). In the presence of O_2_, GOX catalyzes the oxidation of β-*D*-glucose to *D*-glucono-1,5-lactone and H_2_O_2_, which is used by HRP to oxidize an organic molecule. If a nonfluorescent/noncolored HRP substrate is oxidized to a fluorescent/colored species, its detection can be exploited to monitor the progress of the enzymatic cascade by confocal microscopy or visible/fluorescence spectroscopy. The selective and complementary localization of GOX and HRP at dendritic MCM was exploited to assess the efficiency of the enzymatic cascade – including membrane permeability to small molecules (glucose and HRP substrates) and chemical communication (H_2_O_2_) – using a single droplet population coencapsulating both enzymes. The spatially coupled cascade reaction was initially investigated by CLSM experiments. To this end, *o*-phenylenediamine (oPD) was selected as a nonfluorescent HRP substrate, which is oxidized to fluorescent 2,3-diaminophenazine (2,3-DAP) (Figure 4A). Upon addition of glucose to a mixture of oPD and a MCM loaded with GOX-Cy5 (red) and HRP-RITC (cyan) (80 nM GOX and 75 nM HRP), the fluorescent signal of 2,3-DAP (yellow) appeared within minutes at the MCM interior, confirming facile membrane permeability to substrates, rapid enzymatic reactions and intradroplet communication (Figures 4B and 4C). Notably, control experiments carried out under identical conditions in the absence of glucose or any of the enzymes did not produce fluorescence output, confirming the necessity of both proteins for a successful enzymatic cascade (Figure S6).

**Figure 4:**
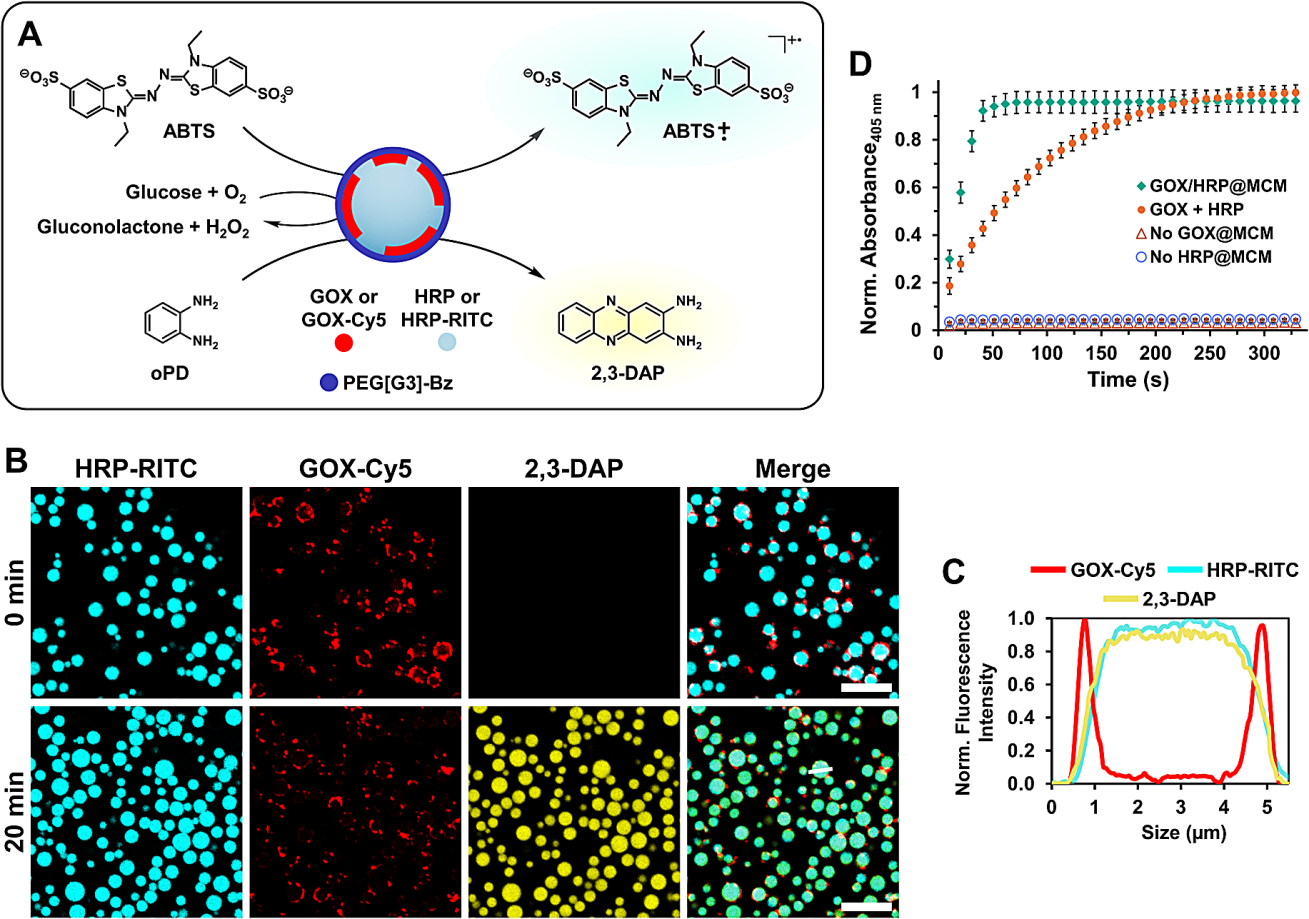
Scheme of the GOX-HRP enzymatic cascade and MCM intradroplet chemical communication (A). CLSM images showing enzymatic production of 2,3-DAP (yellow) by GOX-Cy5/HRP-RITC@MCM (red and cyan, respectively) in the presence of oPD, before (0 min) and after (20 min) addition of glucose. Scale bars 10 μm. (B) Cross-sectional fluorescence intensity profiles (line in B at 20 min) show peripheral localization of GOX-Cy5 and uniform distribution of HRP-RITC and 2,3-DAP within the coacervate interior (C). Time dependence of the cascade reaction monitored using ATBS as HRP substrate by measuring the absorbance of the ABTS radical cation (405 nm) (D).

To analyze the time-dependence of the cascade, 2,2’-azino-bis­(3-ethylbenzothiazoline-6-sulfonic acid) (ABTS) was chosen as HRP substrate (Figure 4A). The one-electron oxidation of ABTS by H_2_O_2_ in the presence of HRP produces the ABTS radical cation, a colored product that absorbs at 405 nm. Continuous measurement of the absorbance increase in a mixture composed of ABTS, GOX/HRP@MCM, and glucose (80 nM GOX and 75 nM HRP) allowed monitoring the reaction progress. Absorbance reached a plateau within 1 min of glucose addition, indicating that the reaction had completed (Figure 4D). No absorbance increase was observed in the absence of glucose or with MCM populations lacking GOX or HRP. Interestingly, when the cascade reaction was performed with free enzymes in solution at the same concentration as in the MCM, absorbance increased more slowly, stabilizing at values comparable to those of the MCM only after more than 4 min (Figure 4D). This significantly more efficient communication for the spatially coupled cascade is likely facilitated by locally increased enzyme and substrate concentrations in the coacervate core.^62,63^ Taken together, these findings demonstrate the ability of dendritic MCM to mimic cell-like behaviors such as enzyme encapsulation, fast internal dynamics, and chemical communication, which make them promising synthetic cell models and bioreactors.

### Communication between Dendritic MCM and Natural Cells: A549 and Red Blood Cells

Despite significant advances in the complexity of synthetic cells over the past decade, communication between artificial and living cells remains a key challenge in synthetic biology.^64–68^ Progress in this area is expected to drive major breakthroughs in advanced therapies, tissue engineering, and regenerative medicine.^69–72^ In particular, enabling synthetic cells to exchange chemical signals with living cells could unlock new opportunities in drug and gene delivery, as well as the development of compartmentalized enzymatic bioreactors.^73,74^ In an effort to integrate synthetic and natural cells, we have explored the potential of dendritic MCM as bioreactors capable of interacting with living cells. As proof of concept, we have selected MCM loaded with GOX (and HRP) as transmitter synthetic cells and human adenocarcinoma alveolar basal epithelial (A549) and red blood cells (RBC) as receiver natural cells (Figures 5 and 6). Interest in the GOX-catalyzed production of H_2_O_2_ stems not only from its role as a model enzymatic reaction for studying communication in synthetic-natural cell consortia,^75–77^ but also as a versatile strategy for multimodal cancer therapy increasing the levels of tumor oxidative stress, with concomitant consumption of glucose and O_2_ (synergistic cancer-starvation and hypoxia-activated therapies).^78^

**Figure 5:**
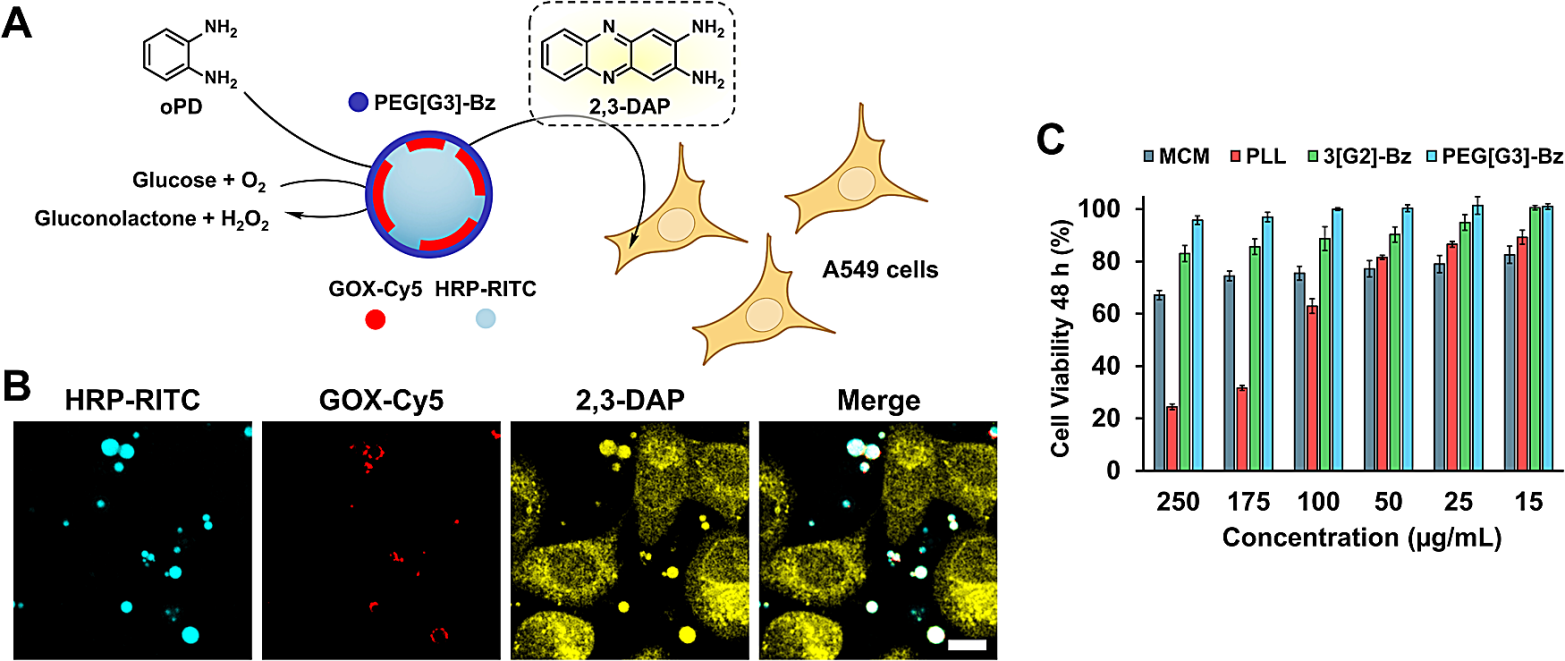
Scheme and CLSM images (20 min) of the enzymatic production of 2,3-DAP in GOX-Cy5/HRP-RITC@MCM and its transfer as a model signaling molecule to A549 cells. Scale bar 10 μm (A, B). Cell viability (CCK-8, 48 h) of A549 cells in the presence of blank MCM and the polyelectrolyte constituents (shown concentrations refer to MCM; 3­[G2]-Bz, PEG­[G3]-Bz, and PLL were used at the same concentrations as in MCM) (C).

**Figure 6:**
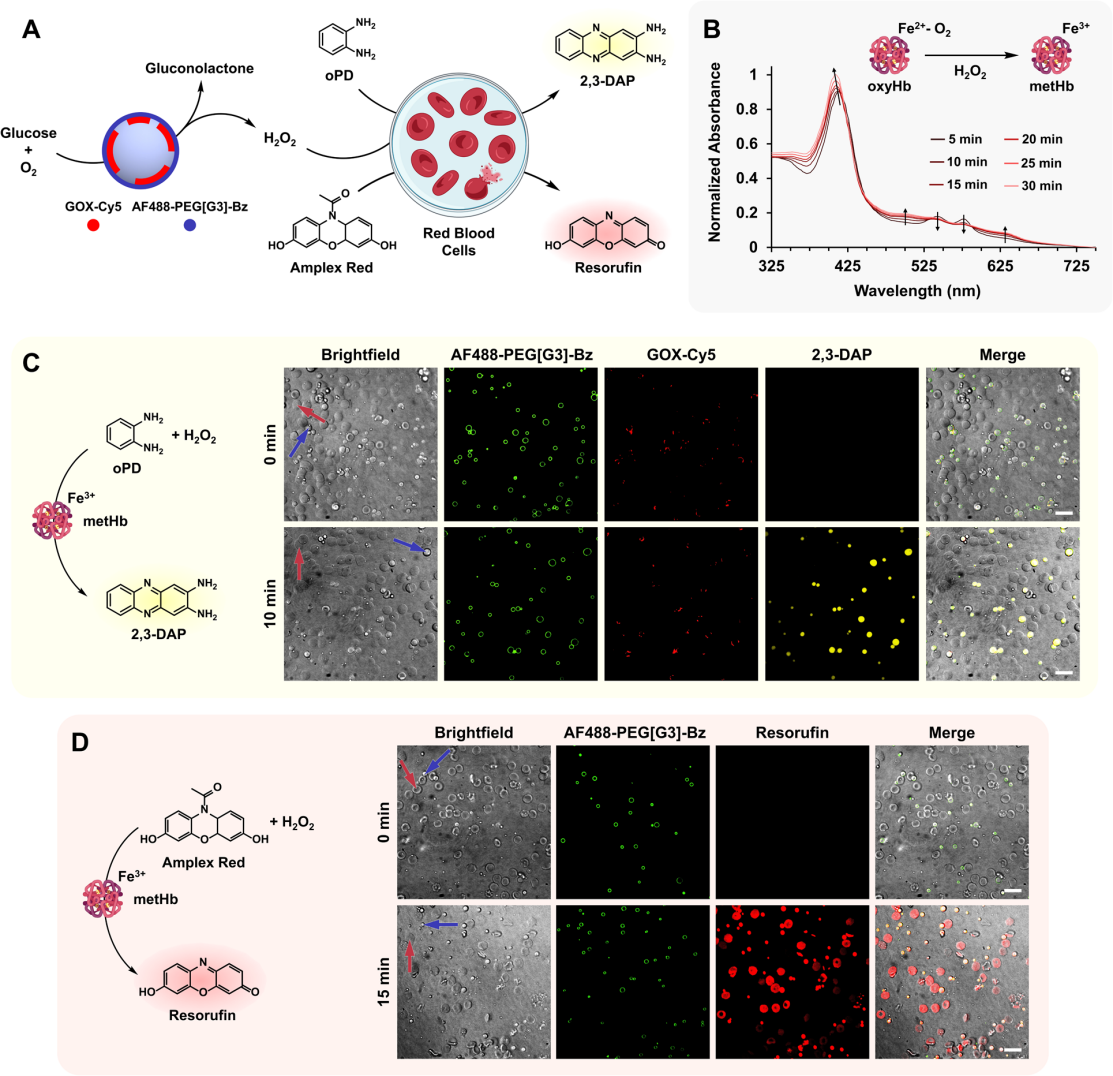
Synthetic-natural cell consortium comprising GOX@MCM and RBC. In response to an extracellular glucose input, communication between synthetic and natural cells (H_2_O_2_ signaling) endows RBC with peroxidase-like activity as confirmed by 2,3-DAP and resorufin fluorescence readouts (A). UV–vis spectroscopy of RBC incubated with GOX@MCM and glucose confirms the oxidation of oxyHb to metHb in response to H_2_O_2_ signaling (B). CLSM and brightfield microscopy images showing enzymatic production of 2,3-DAP (yellow, C) and resorufin (red, D) by GOX-Cy5@MCM (red in C, no color in D) and RBC in the presence of oPD or Amplex Red, before (0 min) and after (10–15 min) addition of glucose. While the small, charged oPD undergoes extracellular oxidation, leading to 2,3-DAP accumulation at dendritic MCM (C), resorufin is also detected inside RBC, as hydrophobic Amplex Red readily diffuses across the cell membrane (D). Scale bars 10 μm. Blue and red arrows indicate individual MCM and RBC, respectively.

A tandem composed of GOX/HRP@MCM and A549 cells was first investigated to study the transfer of a model signaling molecule (2,3-DAP enzymatically produced at the MCM) from synthetic to natural cells (Figure 5A). Prior to the experiments, the cytotoxicity of blank MCM and polyelectrolyte constituents was assessed by CCK-8 assay in A549 cells (Figure 5C). The minimal impact of 3­[G2]-Bz, PEG­[G3]-Bz, and the MCM on cell proliferation, even at the highest concentration analyzed (cell viability close to 70% for MCM after 48 h), contrasts with the well-documented toxicity displayed by PLL, confirming the structural integrity of dendritic MCM under cell culture conditions. Then, A549 cells were incubated with GOX-Cy5/HRP-RITC@MCM (red and cyan by CLSM, respectively) in Dulbecco’s modified Eagle’s medium (DMEM) with high glucose, containing 10% fetal bovine serum. After the addition of oPD to initiate the cascade reaction (80 nM GOX and 75 nM HRP), CLSM confirmed the accumulation of 2,3-DAP (yellow) not only in synthetic but also in living cells with a preferential localization in the cytoplasm and, to a lesser extent, in nuclei (Figure 5B). In control experiments performed in the absence of any of the enzymes or oPD, no 2,3-DAP was observed in A549 cells (Figure S7). These results highlight the potential of dendritic MCM as a synthetic cell platform for the selective delivery of therapeutic enzymes for treating disease through *in situ* production/activation of therapeutics and prodrugs.^79,80^

Next, signal transduction between synthetic and natural cells was investigated using GOX@MCM and RBC (Figure 6A). The strategy pursues endowing mammalian cells with peroxidase activity in response to extracellular glucose via chemical communication (H_2_O_2_ signaling) with a glucose-responsive synthetic cell. The cascade, inspired by earlier work by Mann and co-workers,^81,82^ exploits the peroxidase-like activity of methemoglobin (metHb) produced by H_2_O_2_ oxidation of hemoglobin (Hb) present in RBC. Hb is the major heme protein of RBC and is responsible for the transport of O_2_ to the tissues. The redox state of the heme group of Hb is crucial. While O_2_ binds to the ferrous heme to form oxyhemoglobin (oxyHb), it does not bind to the ferric form of metHb. Interestingly, oxyHb undergoes spontaneous auto-oxidation to metHb at a rate of 3% Hb per day, a process accelerated in the presence of exogenous H_2_O_2_, which diffuses across the RBC membrane very rapidly.^83^ RBC are particularly sensitive to oxidative stress.^84^ The lack of a nucleus or mitochondria limits their ability to repair components, resulting in oxidative damage of the membrane and hemolysis.^85,86^ Despite its primary role as O_2_ carrier, Hb also possesses various enzymatic activities due to its structural similarity to other heme-bearing proteins.^87^ In particular, peroxidase-like activity of metHb has been demonstrated in the H_2_O_2_-dependent oxidation of various organic compounds, including styrene,^88^ *S*-heterocycles,^89,90^ polycyclic aromatic hydrocarbons,^91,92^ oPD,^93,94^ and Amplex Red.^81^ Among these, oPD and Amplex Red, nonfluorescent substrates that are respectively oxidized to the fluorescent products 2,3-DAP and resorufin, were selected as reporters for MCM-to-RBC signal transduction (Figure 6A). Before initiating these experiments, the oxidation of oxyHb to metHb in response to H_2_O_2_ signaling was assessed by incubating a suspension of rat-derived RBC with GOX@MCM in the presence of glucose (Figure 6B). UV–vis spectroscopy confirmed the oxidation process through characteristic changes in the absorbance spectra of the involved species:^95^ a shift of the Soret band from 414 to 404 nm, the disappearance of oxyHb absorbance peaks at 540 and 578 nm, and the emergence of characteristic metHb signals at 500 and 630 nm.

Subsequently, MCM-to-RBC communication experiments were performed to assess the peroxidase-like activity of metHb using oPD and Amplex Red as substrates. The outcome of the cascade reaction was expected to depend on the preferential localization of the substrates and products: within MCM or RBC. Whereas the small, charged oPD was expected to undergo extracellular oxidation by released metHb, leading to 2,3-DAP accumulation within MCM (Figure 6C), the more hydrophobic Amplex Red was anticipated to diffuse across the RBC membrane and be oxidized to resorufin intracellularly (Figure 6D). The cascade between fluorescently labeled GOX-Cy5@MCM and RBC was monitored for both substrates by CLSM and brightfield microscopy following glucose addition (40–45 nM GOX). Visualization of the 2,3-DAP (yellow) and resorufin (red) fluorescence readouts after 10–15 min confirmed efficient communication between synthetic and natural cells – via H_2_O_2_ signaling in response to an extracellular glucose input – and RBC transduction of the signal into peroxidase activity. As expected, a selective localization of the reporters was observed. While 2,3-DAP fluorescence accumulates at dendritic MCM (Figure 6C), resorufin is also detected inside RBC (Figure 6D). Control experiments using a blank MCM (lacking GOX) or in the absence of either GOX-Cy5@MCM or glucose resulted in no detectable fluorescence readout, confirming the necessity of the glucose input and synthetic cell for a successful cascade (Figures S8 and S11). Finally, the time-dependent communication within the synthetic-natural cell consortium was monitored by fluorescence spectroscopy using oPD as the substrate. Continuous measurement of 2,3-DAP fluorescence reached a plateau after approximately 3 h (Figure S9). Overall, our findings underscore the capacity of dendritic MCM to perform enzyme-driven biochemical reactions and to engage in chemical communication with neighboring natural cells, two essential cellular functions for sustaining life.

## Conclusions

Synthetic cells that replicate cellular functions must remain structurally stable in biological environments. For complex coacervates, stability under physiological ionic strength is especially critical. Here, we introduce membranized coacervate microdroplets (MCM) with enhanced salt resistance by employing charged dendrimers – a strategy that leverages the globular, rigid architecture of these tree-like polymers. The complexation of 3­[G2]-Bz (an anionic dendrimer with 27 peripheral carboxylates) and cationic PLL (DP 101) under stoichiometric charge ratio, followed by interfacial stabilization of the resulting coacervate with PEG­[G3]-Bz (a PEG-dendritic block copolymer with 27 carboxylates), affords stable MCM with the copolymer hierarchically assembled at the external interface. The critical salt concentration of dendritic MCM exceeds by more than 2-fold that of coacervates composed of PLL – or branched poly­(ethylenimine) – and anionic polypeptides of much higher degrees of polymerization, highlighting their robustness under physiological conditions and suitability as synthetic cells in biological media. The ability of dendritic MCM to mimic cell-like behavior such as efficient protein encapsulation irrespective of the isoelectric point (GOX, HRP, BSA, lysozyme, insulin), fast internal dynamics (studied by FRAP), and chemical communication (GOX-HRP enzymatic cascade) makes them a promising synthetic cell platform for the selective delivery of therapeutic enzymes for treating disease through *in situ* production/activation of therapeutics and prodrugs. Finally, their ability to engage in signal transduction pathways with neighboring living cells (A549 and RBC), enabling cellular responses to extracellular inputs via chemical signaling, paves their way in tissue engineering and regenerative medicine.

## Supplementary Material



## Notes

Materials, instrumentation, experimental procedures, and characterization (PDF)

## References

[1] Hirschi S., Ward T. R., Meier W. P., Müller D. J., Fotiadis D. (2022). Synthetic Biology: Bottom-Up Assembly of Molecular
Systems. Chem. Rev..

[2] Godoy-Gallardo M., York-Duran M. J., Hosta-Rigau L. (2018). Recent Progress in Micro/Nanoreactors
toward the Creation of Artificial Organelles. Adv. Healthcare Mater..

[3] Palivan C. G., Heuberger L., Gaitzsch J., Voit B., Appelhans D., Borges Fernandes B., Battaglia G., Du J., Abdelmohsen L., van Hest J. C. M., Hu J., Liu S., Zhong Z., Sun H., Mutschler A., Lecommandoux S. (2024). Advancing Artificial Cells with Functional
Compartmentalized Polymeric Systems - In Honor of Wolfgang Meier. Biomacromolecules.

[4] Maffeis V., Heuberger L., Nikoletić A., Schoenenberger C. A., Palivan C. G. (2024). Synthetic Cells Revisited: Artificial
Cell Construction
Using Polymeric Building Blocks. Adv. Sci..

[5] Gozen I., Koksal E. S., Poldsalu I., Xue L., Spustova K., Pedrueza-Villalmanzo E., Ryskulov R., Meng F., Jesorka A. (2022). Protocells:
Milestones and Recent Advances. Small.

[6] Guindani C., Da Silva L. C., Cao S., Ivanov T., Landfester K. (2022). Synthetic
Cells: From Simple Bio-Inspired Modules to Sophisticated Integrated
Systems. Angew. Chem., Int. Ed..

[7] Cook A. B., Novosedlik S., Van Hest J. C. M. (2023). Complex Coacervate
Materials as Artificial
Cells. Acc. Mater. Res..

[8] Ellis R. J. (2001). Macromolecular
crowding: obvious but underappreciated. Trends
Biochem. Sci..

[9] Oparin, A. I. The Origin of Life; 2nd ed.; Dover Publications: 1953.

[10] Hyman T., Brangwynne C. (2012). In Retrospect:
The Origin of Life. Nature.

[11] Sing C. E., Perry S. L. (2020). Recent progress
in the science of complex coacervation. Soft
Matter.

[12] Peng Q., Wang T., Yang D., Peng X., Zhang H., Zeng H. (2024). Recent advances in
coacervation and underlying noncovalent molecular
interaction mechanisms. Prog. Polym. Sci..

[13] Deshpande S., Dekker C. (2021). Studying phase separation
in confinement. Curr. Opin. Colloid Interface
Sci..

[14] Abbas M., Lipiński W. P., Wang J., Spruijt E. (2021). Peptide-based coacervates
as biomimetic protocells. Chem. Soc. Rev..

[15] Lin Z., Beneyton T., Baret J. C., Martin N. (2023). Coacervate Droplets
for Synthetic Cells. Small Methods.

[16] Tang T.-Y. D., Hak C. R. C., Thompson A. J., Kuimova M. K., Williams D. S., Perriman A. W., Mann S. (2014). Fatty acid membrane assembly on coacervate
microdroplets as a step towards a hybrid protocell model. Nat. Chem..

[17] Zhang Y., Chen Y., Yang X., He X., Li M., Liu S., Wang K., Liu J., Mann S. (2021). Giant Coacervate Vesicles
As an Integrated Approach to Cytomimetic Modeling. J. Am. Chem. Soc..

[18] Mason A. F., Buddingh’ B. C., Williams D. S., van Hest J. C. M. (2017). Hierarchical
Self-Assembly of a Copolymer-Stabilized Coacervate Protocell. J. Am. Chem. Soc..

[19] Aumiller W. M., Pir Cakmak F., Davis B. W., Keating C. D. (2016). RNA-Based Coacervates
as a Model for Membraneless Organelles: Formation, Properties, and
Interfacial Liposome Assembly. Langmuir.

[20] Gao N., Xu C., Yin Z., Li M., Mann S. (2022). Triggerable Protocell
Capture in Nanoparticle-Caged Coacervate Microdroplets. J. Am. Chem. Soc..

[21] Leurs Y. H. A., Giezen S. N., Li Y., Van Den Hout W., Beeren J., Van Den Aker L. J. M., Voets I. K., Van Hest J. C. M., Brunsveld L. (2025). Stabilization of Condensate Interfaces Using Dynamic
Protein Insertion. J. Am. Chem. Soc..

[22] Xu C., Martin N., Li M., Mann S. (2022). Living material assembly
of bacteriogenic protocells. Nature.

[23] Gao N., Mann S. (2023). Membranized Coacervate
Microdroplets: from Versatile Protocell Models
to Cytomimetic Materials. Acc. Chem. Res..

[24] Perry S., Li Y., Priftis D., Leon L., Tirrell M. (2014). The Effect of Salt
on the Complex Coacervation of Vinyl Polyelectrolytes. Polymers.

[25] Li L., Srivastava S., Andreev M., Marciel A. B., De Pablo J. J., Tirrell M. V. (2018). Phase Behavior and Salt Partitioning in Polyelectrolyte
Complex Coacervates. Macromolecules.

[26] Caminade, A.-M. ; Turrin, C.-O. ; Laurent, R. ; Ouali, A. ; Delavaux-Nicot, B. Dendrimers: Towards Catalytic, Material and Biomedical Uses; John Wiley & Sons, Ltd: Chichester, U.K., 2011.

[27] Astruc D., Boisselier E., Ornelas C. (2010). Dendrimers Designed for Functions:
From Physical, Photophysical, and Supramolecular Properties to Applications
in Sensing, Catalysis, Molecular Electronics, Photonics, and Nanomedicine. Chem. Rev..

[28] Harada A., Kataoka K. (1995). Formation of Polyion Complex Micelles in an Aqueous
Milieu from a Pair of Oppositely-Charged Block Copolymers with Poly­(ethylene
glycol) Segments. Macromolecules.

[29] Kabanov A. V., Vinogradov S. V., Suzdaltseva Y. G., Alakhov V. Y. (1995). Water-Soluble Block
Polycations as Carriers for Oligonucleotide Delivery. Bioconjugate Chem..

[30] Cohen
Stuart M. A., Besseling N. A. M., Fokkink R. G. (1998). Formation of Micelles
with Complex Coacervate Cores. Langmuir.

[31] Marras A. E., Ting J. M., Stevens K. C., Tirrell M. V. (2021). Advances in the
Structural Design of Polyelectrolyte Complex Micelles. J. Phys. Chem. B.

[32] Zhang G.-D., Nishiyama N., Harada A., Jiang D.-L., Aida T., Kataoka K. (2003). pH-sensitive Assembly of Light-Harvesting
Dendrimer
Zinc Porphyrin Bearing Peripheral Groups of Primary Amine with Poly­(ethylene
glycol)-*b*-poly­(aspartic acid) in Aqueous Solution. Macromolecules.

[33] Sousa-Herves A., Fernandez-Megia E., Riguera R. (2008). Synthesis and supramolecular assembly
of clicked anionic dendritic polymers into polyion complex micelles. Chem. Commun..

[34] Naoyama K., Mori T., Katayama Y., Kishimura A. (2016). Fabrication
of Dendrimer-Based Polyion Complex Submicrometer-Scaled Structures
with Enhanced Stability under Physiological Conditions. Macromol. Rapid Commun..

[35] Fernandez-Villamarin M., Sousa-Herves A., Porto S., Guldris N., Martinez-Costas J., Riguera R., Fernandez-Megia E. (2017). A Dendrimer-Hydrophobic Interaction
Synergy Improves the Stability of Polyion Complex Micelles. Polym. Chem..

[36] Amaral S. P., Tawara M. H., Fernandez-Villamarin M., Borrajo E., Martínez-Costas J., Vidal A., Riguera R., Fernandez-Megia E. (2018). Tuning the Size of Nanoassembles: A Hierarchical Transfer
of Information from Dendrimers to Polyion Complexes. Angew. Chem., Int. Ed..

[37] Lopez-Blanco R., Fernandez-Villamarin M., Jatunov S., Novoa-Carballal R., Fernandez-Megia E. (2019). Polysaccharides meet dendrimers to fine-tune the stability
and release properties of polyion complex micelles. Polym. Chem..

[38] Mignani S., Shi X., Zablocka M., Majoral J.-P. (2021). Dendritic Macromolecular Architectures:
Dendrimer-Based Polyion Complex Micelles. Biomacromolecules.

[39] Lopez-Blanco R., Magana Rodriguez J. R., Esquena J., Fernandez-Megia E. (2025). From nanometric
to giant polyion complex micelles via a hierarchical assembly of dendrimers. J. Colloid Interface Sci..

[40] Pinto L. F., Correa J., Martin-Pastor M., Riguera R., Fernandez-Megia E. (2013). The Dynamics
of Dendrimers by NMR Relaxation: Interpretation Pitfalls. J. Am. Chem. Soc..

[41] Tomalia D. A., Khanna S. N. (2016). A Systematic Framework and Nanoperiodic Concept for
Unifying Nanoscience: Hard/Soft Nanoelements, Superatoms, Meta-Atoms,
New Emerging Properties, Periodic Property Patterns, and Predictive
Mendeleev-like Nanoperiodic Tables. Chem. Rev..

[42] Li L., Deng Y., Zeng Y., Yan B., Deng Y., Zheng Z., Li S., Yang Y., Hao J., Xiao X., Wang X. (2023). The application advances of dendrimers
in biomedical field. View.

[43] Blocher
Mctigue W. C., Perry S. L. (2020). Protein Encapsulation Using Complex
Coacervates: What Nature Has to Teach Us. Small.

[44] Fernandez-Villamarin M., Sousa-Herves A., Correa J., Munoz E. M., Taboada P., Riguera R., Fernandez-Megia E. (2016). The Effect
of PEGylation on Multivalent
Binding: A Surface Plasmon Resonance and Isothermal Titration Calorimetry
Study with Structurally Diverse PEG-Dendritic GATG Copolymers. ChemNanoMat.

[45] Sproncken C. C. M., Magana J. R., Voets I. K. (2021). 100th Anniversary of Macromolecular
Science Viewpoint: Attractive Soft Matter: Association Kinetics, Dynamics,
and Pathway Complexity in Electrostatically Coassembled Micelles. ACS Macro Lett..

[46] Priftis D., Tirrell M. (2012). Phase behaviour and complex coacervation
of aqueous
polypeptide solutions. Soft Matter.

[47] Priftis D., Megley K., Laugel N., Tirrell M. (2013). Complex coacervation
of poly­(ethylene-imine)/polypeptide aqueous solutions: Thermodynamic
and rheological characterization. J. Colloid
Interface Sci..

[48] Perry S. L., Leon L., Hoffmann K. Q., Kade M. J., Priftis D., Black K. A., Wong D., Klein R. A., Pierce C. F., Margossian K. O., Whitmer J. K., Qin J., De Pablo J. J., Tirrell M. (2015). Chirality-selected phase behaviour
in ionic polypeptide
complexes. Nat. Commun..

[49] Cakmak F. P., Choi S., Meyer M. O., Bevilacqua P. C., Keating C. D. (2020). Prebiotically-relevant low polyion multivalency can
improve functionality of membraneless compartments. Nat. Commun..

[50] Vieregg J. R., Lueckheide M., Marciel A. B., Leon L., Bologna A. J., Rivera J. R., Tirrell M. V. (2018). \. J. Am. Chem.
Soc..

[51] Mignani S., El Kazzouli S., Bousmina M. M., Majoral J.-P. (2014). Dendrimer Space
Exploration: An Assessment of Dendrimers/Dendritic Scaffolding as
Inhibitors of Protein–Protein Interactions, a Potential New
Area of Pharmaceutical Development. Chem. Rev..

[52] Shcharbin D., Shcharbina N., Dzmitruk V., Pedziwiatr-Werbicka E., Ionov M., Mignani S., de la Mata F. J., Gómez R., Muñoz-Fernández M. A., Majoral J.-P., Bryszewska M. (2017). Dendrimer-protein interactions versus
dendrimer-based nanomedicine. Colloid Surface
B.

[53] Lippincott-Schwartz J., Snapp E. L., Phair R. D. (2018). The Development
and Enhancement of
FRAP as a Key Tool for Investigating Protein Dynamics. Biophys. J..

[54] Luby-Phelps K., Lanni F., Taylor D. L. (1985). Behavior of a fluorescent
analogue
of calmodulin in living 3T3 cells. J. Cell Biol..

[55] Elowitz M. B., Surette M. G., Wolf P.-E., Stock J. B., Leibler S. (1999). Protein Mobility
in the Cytoplasm of *Escherichia coli*. J. Bacteriol..

[56] Hinow P., Rogers C. E., Barbieri C. E., Pietenpol J. A., Kenworthy A. K., DiBenedetto E. (2006). The DNA Binding Activity of p53 Displays
Reaction-Diffusion Kinetics. Biophys. J..

[57] Abu-Arish A., Porcher A., Czerwonka A., Dostatni N., Fradin C. (2010). High Mobility
of Bicoid Captured by Fluorescence Correlation Spectroscopy: Implication
for the Rapid Establishment of Its Gradient. Biophys. J..

[58] Stasevich T. J., Mueller F., Michelman-Ribeiro A., Rosales T., Knutson J. R., McNally J. G. (2010). Cross-Validating FRAP and FCS to Quantify the Impact
of Photobleaching on In Vivo Binding Estimates. Biophys. J..

[59] Persson L. B., Ambati V. S., Brandman O. (2020). Cellular Control of Viscosity Counters
Changes in Temperature and Energy Availability. Cell.

[60] Spruijt E., Cohen Stuart M. A., Van Der Gucht J. (2013). Linear Viscoelasticity of Polyelectrolyte
Complex Coacervates. Macromolecules.

[61] Fisher R. S., Elbaum-Garfinkle S. (2020). Tunable multiphase
dynamics of arginine and lysine
liquid condensates. Nat. Commun..

[62] Spruijt E. (2023). Open questions
on liquid–liquid phase separation. Commun.
Chem..

[63] Harris R., Berman N., Lampel A. (2025). Coacervates as enzymatic
microreactors. Chem. Soc. Rev..

[64] Gardner P. M., Winzer K., Davis B. G. (2009). Sugar synthesis
in a protocellular
model leads to a cell signalling response in bacteria. Nat. Chem..

[65] Lentini R., Santero S. P., Chizzolini F., Cecchi D., Fontana J., Marchioretto M., Del Bianco C., Terrell J. L., Spencer A. C., Martini L., Forlin M., Assfalg M., Serra M. D., Bentley W. E., Mansy S. S. (2014). Integrating artificial with natural
cells to translate chemical messages that direct E. coli behaviour. Nat. Commun..

[66] Toparlak Ö. D., Zasso J., Bridi S., Serra M. D., Macchi P., Conti L., Baudet M.-L., Mansy S. S. (2020). Artificial cells
drive neural differentiation. Sci. Adv..

[67] Yao Y., Zhang Y., Li L., Huang Y., Yang X., Peng Z., Wang K., Liu J. (2021). Photothermally Activated
Coacervate Model Protocells as Signal Transducers Endow Mammalian
Cells with Light Sensitivity. Adv. Biol..

[68] De
Luis B., Morellá-Aucejo Á., Llopis-Lorente A., Martínez-Latorre J., Sancenón F., López C., Murguía J. R., Martínez-Máñez R. (2022). Nanoprogrammed
Cross-Kingdom Communication Between Living Microorganisms. Nano Lett..

[69] Elani Y. (2021). Interfacing
Living and Synthetic Cells as an Emerging Frontier in Synthetic Biology. Angew. Chem., Int. Ed..

[70] Mukwaya V., Mann S., Dou H. (2021). Chemical communication at the synthetic
cell/living cell interface. Commun. Chem..

[71] Valente S., Galanti A., Maghin E., Najdi N., Piccoli M., Gobbo P. (2024). Matching Together Living Cells and
Prototissues: Will There Be Chemistry?. ChemBioChem.

[72] Meng H., Ji Y., Qiao Y. (2025). Interfacing Complex
Coacervates with Natural Cells. ChemSystemsChem.

[73] Jiang W., Wu Z., Gao Z., Wan M., Zhou M., Mao C., Shen J. (2022). Artificial Cells: Past,
Present and Future. ACS Nano.

[74] Xu Q., Zhang Z., Lui P. P. Y., Lu L., Li X., Zhang X. (2023). Preparation and biomedical
applications of artificial cells. Mater. Today
Bio.

[75] Elani Y., Trantidou T., Wylie D., Dekker L., Polizzi K., Law R. V., Ces O. (2018). Constructing vesicle-based artificial
cells with embedded living cells as organelle-like modules. Sci. Rep..

[76] Wang X., Tian L., Du H., Li M., Mu W., Drinkwater B. W., Han X., Mann S. (2019). Chemical communication
in spatially organized protocell colonies and protocell/living cell
micro-arrays. Chem. Sci..

[77] Zhang Y., Liu S., Yao Y., Chen Y., Zhou S., Yang X., Wang K., Liu J. (2020). Invasion and Defense Interactions
between Enzyme-Active Liquid Coacervate Protocells and Living Cells. Small.

[78] Fu L.-H., Qi C., Lin J., Huang P. (2018). Catalytic chemistry of glucose oxidase
in cancer diagnosis and treatment. Chem. Soc.
Rev..

[79] Fejerskov B., Jarlstad
Olesen M. T., Zelikin A. N. (2017). Substrate mediated enzyme prodrug
therapy. Adv. Drug Delivery Rev..

[80] Kumar R., Kaur C., Kaur K., Khurana N., Singh G. (2023). Prodrugs:
Harnessing chemical modifications for improved therapeutics. J. Drug Del. Sci. Technol..

[81] Wang X., Tian L., Ren Y., Zhao Z., Du H., Zhang Z., Drinkwater B. W., Mann S., Han X. (2020). Chemical Information
Exchange in Organized Protocells and Natural Cell Assemblies with
Controllable Spatial Positions. Small.

[82] Liu S., Zhang Y., Li M., Xiong L., Zhang Z., Yang X., He X., Wang K., Liu J., Mann S. (2020). Enzyme-mediated nitric oxide production in vasoactive erythrocyte
membrane-enclosed coacervate protocells. Nat.
Chem..

[83] Möller M. N., Orrico F., Villar S. F., López A. C., Silva N., Donzé M., Thomson L., Denicola A. (2023). Oxidants and
Antioxidants in the Redox Biochemistry of Human Red Blood Cells. ACS Omega.

[84] Orrico F., Laurance S., Lopez A. C., Lefevre S. D., Thomson L., Möller M. N., Ostuni M. A. (2023). Oxidative Stress
in Healthy and Pathological
Red Blood Cells. Biomolecules.

[85] Quaye I. K. (2015). Extracellular
hemoglobin: the case of a friend turned foe. Front. Physiol..

[86] Rifkind J. M., Mohanty J. G., Nagababu E. (2015). The pathophysiology
of extracellular
hemoglobin associated with enhanced oxidative reactions. Front. Physiol..

[87] Giardina B. (2022). Hemoglobin:
Multiple molecular interactions and multiple functions. An example
of energy optimization and global molecular organization. Mol. Aspects Med..

[88] Ortiz
de Montellano P. R., Catalano C. E. (1985). Epoxidation of styrene by hemoglobin
and myoglobin. Transfer of oxidizing equivalents to the protein surface. J. Biol. Chem..

[89] Alvarez J. C., Ortiz De Montellano P.
R. (1992). Thianthrene 5-oxide
as a probe of
the electrophilicity of hemoprotein oxidizing species. Biochemistry.

[90] Klyachko N. L., Klibanov A. M. (1992). Oxidation of Dibenzothiophene Catalyzed by Hemoglobin
and Other Hemoproteins in Various Aqueous-Organic Media. Appl. Biochem. Biotechnol..

[91] Ortizleon M., Velasco L., Vazquezduhalt R. (1995). Biocatalytic Oxidation of Polycyclic
Aromatic Hydrocarbons by Hemoglobin and Hydrogen Peroxide. Biochem. Biophys. Res. Commun..

[92] Keum H., Kim J., Joo Y. H., Kang G., Chung N. (2021). Hemoglobin peroxidase
reaction of hemoglobin efficiently catalyzes oxidation of benzo­[*a*]­pyrene. Chemosphere.

[93] Zhang K., Cai R., Chen D., Mao L. (2000). Determination of hemoglobin based
on its enzymatic activity for the oxidation of o-phenylenediamine
with hydrogen peroxide. Anal. Chim. Acta.

[94] Li D.-J., Li X.-W., Xie Y.-X., Cai X.-Q., Zou G.-L. (2005). Identification
of intermediate and product from methemoglobin-catalyzed oxidation
of o-phenylenediamine in two-phase aqueous?organic system. Biochemistry.

[95] Ledvina M. (1987). Rapid spectrophotometric
determination of carbonylhemoglobin in blood. Biochem. Clin. Bohemoslov..

