# Supplementary material for: Dendritic Membranized Coacervate Microdroplets: A Robust Platform for Synthetic-Living Cell Consortia
Source: J Am Chem Soc. 2025 Aug 2;147(32):29457–67. doi: 10.1021/jacs.5c09772 (PMC12356591; doi:10.1021/jacs.5c09772)
Supplement: Supplementary file 1 [file ja5c09772_si_001.pdf]

Supporting Information for:

Dendritic Membranized Coacervate Microdroplets: A Robust Platform for Synthetic-Living Cell Consortia

*Celia Jimenez-Lopez, Lucas Garcia-Abuin, and Eduardo Fernandez-Megia**

Centro Singular de Investigación en Química Biolóxica e Materiais Moleculares (CiQUS), Departamento de Química Orgánica, Universidade de Santiago de Compostela, Jenaro de la Fuente s/n, 15782 Santiago de Compostela, Spain.

Table of Contents

1. Materials S3

2. Instrumentation S4

3. Fluorescent Labelling of Compounds S5

4. Preparation and Characterization of Membranized Coacervate Microdroplets (MCM) S9

5. Protein Encapsulation (General Procedure II) S13

6. Transfer of Macromolecular Components Between Dendritic MCM Populations S16

7. Internal Dynamics within Dendritic MCM. FRAP Measurements S17

8. Intra-MCM GOX-HRP Enzymatic Cascade Reaction S19

9. Synthetic Cell-Natural Cell Communication Assays: A549 S22

10. Synthetic Cell-Natural Cell Communication Assays: Red Blood Cells (RBC) S25

11. References S30

1. Materials

Dulbeco´s modified Eagle´s medium (DMEM), fetal bovine serum (FBS), penicillin and streptomycin were purchased from Invitrogen Thermo Fisher. 2,2′-Azino-bis(3-ethylbenzothiazoline-6-sulfonic acid) (ABTS) was purchased from Alfa Aesar. *N*-Acetyl-3,7-dihydroxyphenoxazine (Amplex Red) was supplied by Biosynth. Glucose oxidase (GOX) from *Aspergiullus niger*, peroxidase from horseradish type VI (HRP), bovine serum albumin fraction V (BSA), human recombinant insulin, and lysozyme from chicken egg white were supplied by Sigma-Aldrich. All other chemicals were purchased from Acros Organics, Fluka, or Thermo Fisher Scientific, unless otherwise noted. All solvents were HPLC grade, purchased from Scharlab, Sigma-Aldrich or Acros Organics. DMF was dried using a SPS800 solvent purification system from MBRAUN. H_2O of Mili-Q grade was obtained using a Millipore water purification system. PEG[G3]-Bz, AF488-PEG[G3]-Bz, BocHN-PEG[G3]-N_3, and 3[G2]-Bz were prepared following procedures previously described by our group.[1,2] Poly-L-lysine hydrobromide PLL (M_n 21300, DP 101 by SEC-MALLS) was purchased from Polypeptide Therapeutic Solutions. Cy5-NHS and AF488-NHS were purchased from Lumiprobe GmbH. Rhodamine B isothiocyanate was obtained from Sigma-Aldrich.

2. Instrumentation

Determination of pH Values. pH values were measured with a portable pH-meter (Crison PH25) connected to a glass electrode (Crison 52 09).

Dialysis and Ultrafiltration. Dialysis was performed with an 18 mm Spectra/Por 6 MWCO 1 kDa membrane tubing from SpectrumLabs. Purifications by ultrafiltration were performed on Millipore Amicon stirred cells with Amicon YM3 regenerated cellulose membranes (MWCO 3 kDa) under a 5 psi N_2 pressure.

UV-Vis Spectroscopy. UV-Vis spectra were recorded on a Jasco V-630 spectrometer.

Fluorescence Spectroscopy. Fluorescence measurements were performed in a plate reader Tecan Infinite F200 PRO. Gain was fixed or selected as optimal for dynamic and static measurements, respectively.

Confocal Laser Scanning Microscopy (CLSM). Confocal images were captured on an Andor Dragonfly spinning disk confocal system mounted on a Nikon TiE microscope equipped with a Zyla 4.2 PLUS sCMOS digital camera (Andor, Oxford Instruments). Samples were excited with four different lasers (405, 488, 561, and 637 nm) and the emitted fluorescence was collected by the filter wheel (450/50 nm, 525/50 nm, 620/50 nm, and 725/40 nm) with appropriate combinations of them. Images were taken with a 100× magnification objective. All images were processed with ImageJ software (version 1.51j8).

Fluorescence Recovery After Photobleaching (FRAP). FRAP experiments were performed on an inverted CLSM Leica Stellaris 8 FALCON (Leica Microsystems, Wetzlar, Germany) employing the FRAP interface available in Leica Application Suite X (LAS X) software.

3. Fluorescent Labelling of Compounds

Fluorescent Labelling of Polymers

AF488-PEG[G3]-Bz. A solution of AF488-NHS (4.2 mg, 5.70 μmol, 10 eq) in DMSO (150 μL) was added to a solution of H_2N-PEG[G3]-Bz (10.0 mg, 0.57 μmol) in DMF (2 mL). After stirring overnight at rt under Ar protected from light, the reaction mixture was purified by ultrafiltration (6 × 15 mL 0.1 M $NaHCO_3$ pH 9.0, 3 × 15 mL H_2O; YM3) and freeze-dried to afford AF488-PEG[G3]-Bz as a pale orange solid (8.8 mg, 88%). A degree of functionalization of 50% in AF488 was determined by absorbance at 495 nm (ε_{495}: 71800 $cm^{-1}M^{-1}$ as provided by supplier).

H_2N-PEG[G3]-N_3. BocHN-PEG[G3]-N_3 (25.0 mg, 2.07 μmol) was dissolved in MeOH (392 μL) and 37% HCl (130 μL). After 90 min of stirring at rt, the mixture was concentrated, dissolved in sat $NaHCO_3$, and purified by ultrafiltration (YM3) washing with H_2O. After freeze-drying, H_2N-PEG[G3]-N_3 was obtained (25 mg, 92%) as a pale yellow foam.

AF488-PEG[G3]-N_3. A solution of AF488-NHS (5.7 mg, 7.35 μmol, 5 eq) in DMSO (300 μL) was added to a solution of H_2N-PEG[G3]-N_3 (20.0 mg, 1.47 μmol) in DMF (4 mL). After stirring overnight at rt under Ar protected from light, the reaction mixture was purified by ultrafiltration (6 × 20 mL 0.1 M $NaHCO_3$ pH 9.0, 3 × 20 mL H_2O; YM3) and freeze-dried to afford AF488-PEG[G3]-N_3 as a pale orange solid (18.5 mg, 91%). A degree of functionalization of 35% in AF488 was determined by absorbance at 495 nm (ε_{495}: 71800 $cm^{-1}M^{-1}$ as provided by supplier).

AF488-PEG[G3]-NH$_2$. Ph_3P (6.3 mg, 24 µmol) was added to a solution of AF488-PEG[G3]-N_3 (10.0 mg, 0.72 µmol) in acetone/H_2O (10:1, 0.2 mL). After 18 h of stirring at rt, 3 M HCl (13 µL) was added and the solvent was evaporated. The crude product was dissolved in H_2O (30 mL), filtered through a cotton plug, and washed with $CHCl_3$ (3 x 30 mL). The aqueous phase was dialyzed against 10 mM PB pH 7.4, 150 mM NaCl and kept in the fridge as a ready to use solution for coacervate membranization.

PLL-Cy5. A solution of Cy5-NHS (0.13 mg, 0.18 µmol, 1 eq) in DMSO (20 µL) was added to a solution of PLL (3.80 mg, 0.18 µmol) in 0.1 M $NaHCO_3$ pH 9.0 (1.90 mL). After 18 h of stirring at rt under Ar protected from light, the reaction mixture was purified by dialysis (2 × 500 mL H_2O, 3 × 500 mL 10 mM NaH_2PO_4, 3 × 500 mL 150 mM NaBr, 4 × 500 mL H_2O; MWCO 1 kDa) and freeze-dried to afford PLL-Cy5 as a blue solid (3.20 mg, 84%). A degree of functionalization of 44% in Cy5 was determined by absorbance at 640 nm (ε_{640}: 250000 $cm^{-1}M^{-1}$ as provided by supplier).

PLL-AF488. A solution of AF488-NHS (0.07 mg, 0.09 µmol, 0.5 eq) in DMSO (20 µL) was added to a solution of PLL (4.0 mg, 0.19 µmol) in 0.1 M $NaHCO_3$ pH 9.0 (2 mL). After 18 h of stirring at rt under Ar protected from light, the reaction mixture was purified by dialysis (2 × 500 mL H_2O, 3 × 500 mL 10 mM NaH_2PO_4, 3 × 500 mL 150 mM NaBr, 4 × 500 mL H_2O; MWCO 1 kDa) and freeze-drying to afford PLL-AF488 as a yellow solid (3.70 mg, 92%). A degree of functionalization of 21% in AF488 was determined by absorbance at 495 nm (ε_{495}: 71800 $cm^{-1}M^{-1}$ as provided by supplier).

Fluorescent Labelling of Proteins (General Procedure I)

Proteins were dissolved at 5 mg/mL in 0.1 M $NaHCO_3$ pH 9.0. For insulin, a solution was prepared by adding 0.1 M $NaHCO_3$ (1.5 mL) to insulin (5.5 mg, 0.93 μmol) followed by 0.12 M HCl (60 μL). Then, 0.1 M NaOH (70 μL) was added and the final volume was adjusted to 2 mL, achieving a final insulin concentration of 2.75 mg/mL and pH 9.0. Then, a fresh solution of fluorescent dye in DMSO was added and the reaction mixture was stirred overnight at rt protected from light. After freeze-drying, the crude product was dissolved in 150 μL of 300 mM NaCl and purified in a PD-10 column (Sephadex G-25 resin) to remove the free dye. Fractions of pure fluorescently labelled proteins were desalted by dialysis (5 × 500 mL H_2O; MWCO 1 kDa) and lyophilized. The dye functionalization degrees were determined by measuring the relative UV absorbances of the proteins and fluorescent dyes using the following extinction coefficients: GOX ε_{280} 267200 $cm^{-1}M^{-1}$, HRP ε_{405} 102000 $cm^{-1}M^{-1}$, BSA ε_{280} 43824 $cm^{-1}M^{-1}$, Insulin ε_{280} 5734 $cm^{-1}M^{-1}$, Lysozyme ε_{280} 37970 $cm^{-1}M^{-1}$, Cy5 ε_{640} 250000 $cm^{-1}M^{-1}$, RITC ε_{560} 106000 $cm^{-1}M^{-1}$.

GOX-Cy5. From a solution of Cy5-NHS (0.75 mg, 1.13 μmol, 20 eq) in DMSO (80 μL) and a solution of GOX (9.0 mg, 0.06 μmol) in 0.1 M $NaHCO_3$ (1.8 mL), GOX-Cy5 (8.5 mg, 94%) labelled with an average of 2.1 molecules of Cy5 was obtained following General Procedure I.

HRP-Cy5. From a solution of Cy5-NHS (1.09 mg, 1.64 μmol, 24 eq) in DMSO (80 μL) and a solution of HRP (3.0 mg, 0.07 μmol) in 0.1 M $NaHCO_3$ (0.6 mL), HRP-Cy5 (2.8 mg, 93%) labelled with an average of 2.2 molecules of Cy5 was obtained following General Procedure I.

HRP-RITC. From a solution of rhodamine B isothiocyanate (0.34 mg, 0.64 μmol, 10 eq) in DMSO (30 μL) and a solution of HRP (2.8 mg, 0.06 μmol) in 0.1 M $NaHCO_3$ (0.6 mL), HRP-RITC (2.5 mg, 89%) labelled with an average of 1.7 molecules of RITC was obtained following General Procedure I.

BSA-Cy5. From a solution of Cy5-NHS (1.12 mg, 1.68 μmol, 12 eq) in DMSO (100 μL) and a solution of BSA (9.0 mg, 0.14 μmol) in 0.1 M $NaHCO_3$ (1.8 mL), BSA-Cy5 (8.1 mg, 90%) labelled with an average of 0.9 molecules of Cy5 was obtained following General Procedure I.

Insulin-Cy5. From a solution of Cy5-NHS (5.50 mg, 8.24 μmol, 12 eq) in DMSO (80 μL) and a solution of insulin (4.1 mg, 0.71 μmol) in 0.1 M $NaHCO_3$ (1.5 mL), insulin-Cy5 (3.6 mg, 88%) labelled with an average of 0.9 molecules of Cy5 was obtained following General Procedure I.

Lysozyme-Cy5. From a solution of Cy5-NHS (3.59 mg, 5.38 μmol, 11 eq) in DMSO (80 μL) and a solution of lysozyme (7.5 mg, 0.51 μmol) in 0.1 M $NaHCO_3$ (1.5 mL), lysozyme-Cy5 (6.8 mg, 91%) labelled with an average of 1.8 molecules of Cy5 was obtained following General Procedure I.

4. Preparation and Characterization of Membranized Coacervate Microdroplets (MCM)

Preparation of MCM. PEG[G3]-Bz (3.08 mg/mL), AF488-PEG[G3]-Bz (3.11 mg/mL), and 3[G2]-Bz (2.16 mg/mL) were separately dissolved in 10 mM Na_2HPO_4/0.1 M NaOH (15% v/v), 150 mM NaCl. PLL (0.50 mg/mL), PLL-Cy5 (0.51 mg/mL), and PLL-AF488 (0.50 mg/ml) were separately dissolved in 10 mM NaH_2PO_4, 150 mM NaCl. These solutions were aged at rt overnight before preparation of coacervates.

Coacervates were prepared by adding a solution of PLL (200 µL) to a solution of 3[G2]-Bz (100 µL), maintaining a stoichiometric ratio of amines and carboxylates. After 40 min of orbital stirring (350 rpm) in an Eppendorf Thermomixer C at 21 °C, a solution of PEG[G3]-Bz (9 µL, equivalent to 9 mol% of 3[G2]-Bz) was added to interfacially stabilize the coacervates and afford membranized coacervate microdroplets (MCM). The final pH of the dispersion was 7.3-7.5. Identical experimental conditions were followed for the preparation of fluorescently labelled MCM using previously prepared solutions of PEG[G3]-Bz/AF488-PEG[G3]-Bz (molar ratio 10:1), PLL/PLL-Cy5 (molar ratio 10:1), or PLL/PLL-AF488 (molar ratio 6.7:1). The presence of spherical droplets and the absence of aggregates was confirmed by optical microscopy and CLSM. MCM remained stable for at least 48 h. The size distribution of the MCM was determined by measuring the diameter of 140 droplets in optical microscopy images using ImageJ software.

Turbidimetry. Turbidity measurements were performed on a Jasco V-630 UV-Vis spectrophotometer at a wavelength of 500 nm. Samples were prepared by diluting 50 µL of MCM suspension with 50 µL of NaCl solutions of increasing concentration. Samples were then placed in a cuvette with a 1 cm path length, and turbidity (T) at 25 °C was determined using Eq S1, where A is the absorbance.

$$T\ (\%) = 100 - 10^{(2-A)} \qquad \text{Eq S1}$$

For CLSM studies, each MCM sample was transferred to a microscope slide (Cellvis 4-Chamber microwells 35 mm Glass Bottom Dish with 20 mm, #1.5 cover glass) for analysis.

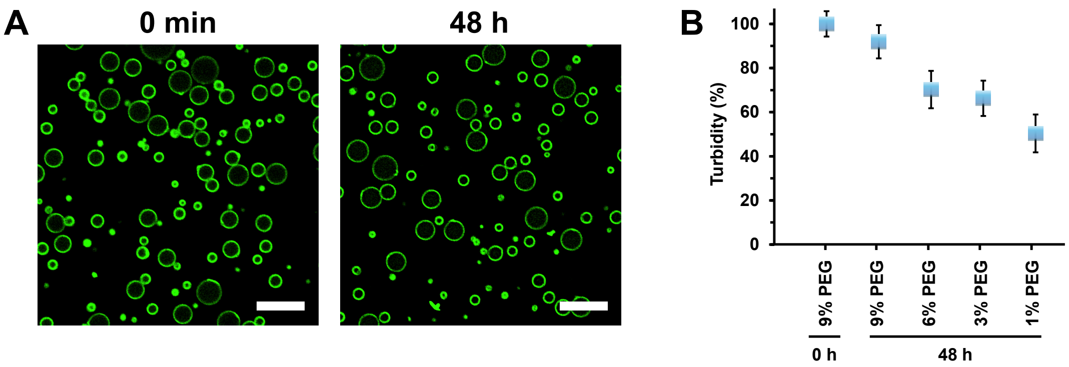

Figure S1. CLSM images of MCM stabilized with 9 mol% AF488-PEG[G3]-Bz (green) immediately after preparation and 48 h later. Scale bars 10 µm (**A**). Turbidity of MCM stabilized with different amounts of PEG[G3]-Bz (1, 3, 6, and 9 mol% relative to 3[G2]-Bz) recorded 48 h after preparation. Comparison with a freshly prepared MCM containing 9 mol% PEG[G3]-Bz (**B**).

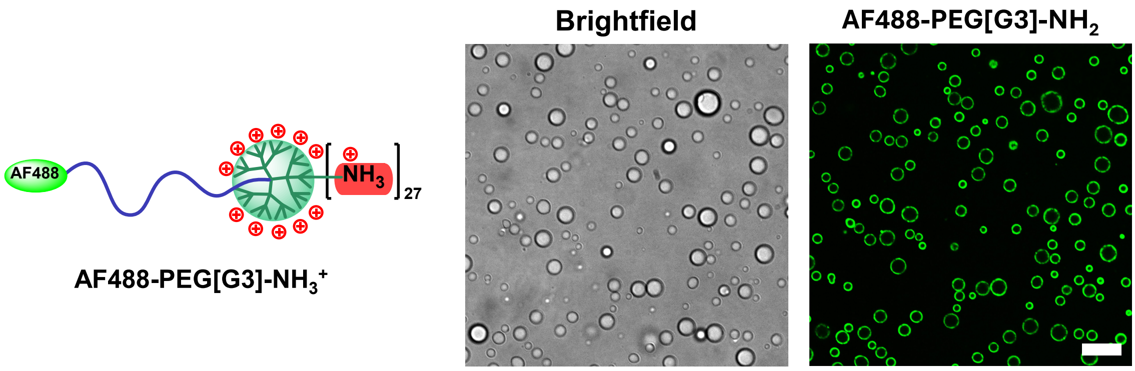

Figure S2. CLSM and brightfield microscopy images of dendritic MCM interfacially stabilized with 3 mol% AF488-PEG[G3]-NH_2·HCl (green) hierarchically assembled at the external interface of the droplets. Scale bar 10 µm.

Size Modulation of MCM. MCM at increasing concentrations were prepared to modulate the size of the droplets. Solutions of 3[G2]-Bz and PLL were prepared at the concentrations shown in Table S1 and allowed to age at rt overnight. Coacervates were prepared as described above by adding PLL (200 µL) to 3[G2]-Bz (100 µL), maintaining a stoichiometric ratio of amines and carboxylates. After 40 min of orbital stirring, a solution of AF488-PEG[G3]-Bz/PEG[G3]-Bz (12 mg/mL) was added to the coacervate suspension. The size distribution of the resulting MCM was determined by measuring the diameter of 100 to 140 droplets in optical microscopy images using ImageJ software. Size distributions were plotted as histograms using Origin 2022 software (OriginLab) (Figure S3).

Table S1. Concentration of 3[G2]-Bz and PLL solutions and volume of AF488-PEG[G3]-Bz/PEG[G3]-Bz solution used in the preparation of MCM with increasing concentrations.

	**3[G2]-Bz (mg/mL)**	**PLL (mg/mL)**	**AF488-PEG[G3]-Bz / PEG[G3]-Bz (μL)**	**MCM (mg/mL)**	**Size (μm)**
**1x**	2.16	0.50	2.28	1.11	3.60 ± 1.02
**4x**	8.65	2.00	8.92	4.43	4.20 ± 1.08
**6x**	13.02	3.00	13.43	6.58	4.70 ± 1.20
**8x**	17.65	4.00	17.91	8.72	5.25 ± 1.16

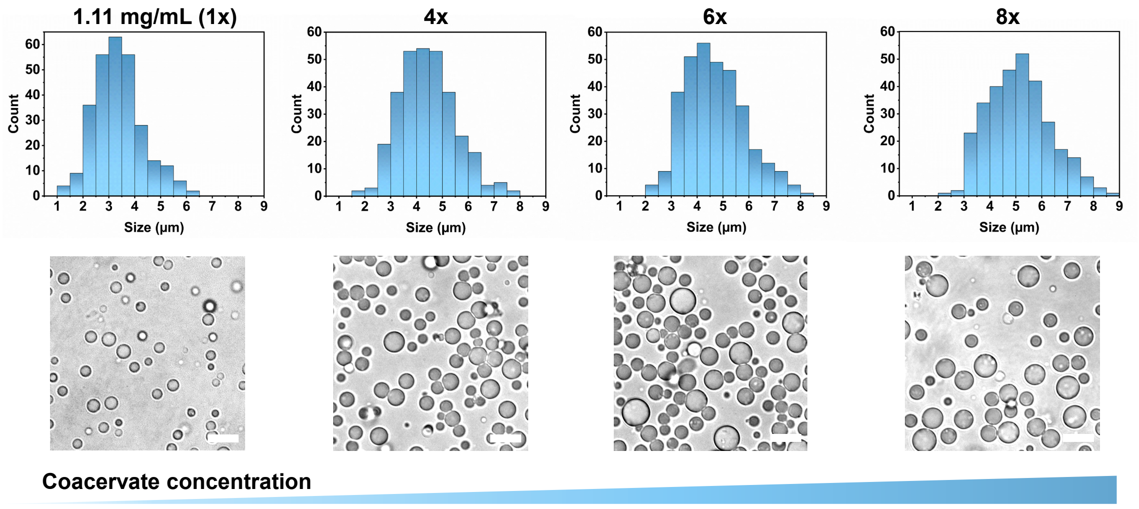

Figure S3. Size distributions and brightfield microscopy images of MCM prepared at increasing polymer concentrations. Scale bars 10 μm.

5. Protein Encapsulation (General Procedure II)

A solution of protein was added to the coacervate mixture immediately after mixing PLL (200 μL) and 3[G2]-Bz (100 μL). The resulting mixture was left under orbital stirring (350 rpm) at 21 °C in an Eppendorf Thermomixer C. After 40 min, a solution of AF488-PEG[G3]-Bz/PEG[G3]-Bz (9 μL) was added, and enzyme encapsulation was confirmed by CLSM. The sample was centrifuged at 12000 g for 5 min to remove unencapsulated protein. Encapsulation efficiency (EE) was determined by comparing the fluorescence of the supernatant with a standard calibration curve made from the fluorescence of fresh enzyme solutions of known concentrations prepared under identical conditions. Before performing further experiments, MCM were resuspended (orbital stirring, 350 rpm, 5 min) in 10 mM PB pH 7.4, 150 mM NaCl.

GOX-Cy5@MCM was prepared from a solution of GOX/GOX-Cy5 (6.32 μL, 2 mg/mL in 10 mM PB pH 7.4, 150 mM NaCl; molar ratio 6.25:1) following General Procedure II. An EE of 82% was determined for GOX-Cy5.

HRP-Cy5@MCM was prepared from a solution of HRP/HRP-Cy5 (2.50 μL, 2 mg/mL in 10 mM PB pH 7.4, 150 mM NaCl; molar ratio 3.33:1) following General Procedure II. An EE of 76% was determined for HRP-Cy5.

GOX-Cy5/HRP-RITC@MCM. Solutions of GOX/GOX-Cy5 (2 mg/mL, molar ratio 6.25:1) and HRP/HRP-RITC (2 mg/mL, molar ratio 3.33:1) in 10 mM PB pH 7.4, 150 mM NaCl were prepared. Enzyme encapsulation was done by adding 2.50 μL of the HRP/HRP-RITC solution to the PLL solution (200 μL). After 1 min, this mixture was added to the 3[G2]-Bz solution (100 μL), followed by immediate addition of 6.32 μL of the GOX/GOX-Cy5 solution. The resulting mixture was left under orbital stirring (350 rpm) at 21 °C in an Eppendorf Thermomixer C. After 40 min, a solution of

AF488-PEG[G3]-Bz/PEG[G3]-Bz (9 µL) was added, and enzyme encapsulation was confirmed by CLSM. The sample was centrifuged at 12000 g for 5 min to remove unencapsulated proteins. EE were determined by comparing the fluorescence of the supernatant with standard calibration curves made from the fluorescence of fresh enzyme solutions of known concentrations prepared under identical conditions. An EE of 67% was determined for HRP-RITC and 76% for GOX-Cy5.

BSA-Cy5@MCM was prepared from a solution of BSA-Cy5 (3.16 µL, 2 mg/mL in 10 mM PB pH 7.4, 150 mM NaCl) following General Procedure II. An EE of 82% was determined for BSA-Cy5.

Insulin-Cy5@MCM was prepared from a solution of insulin/insulin-Cy5 (6.32 µL, 2 mg/mL in 10 mM PB pH 7.4, 150 mM NaCl; molar ratio 2.33:1) following General Procedure II. An EE of 71% was determined for insulin-Cy5.

Lysozyme-Cy5@MCM was prepared from a solution of lysozyme/lysozyme-Cy5 (2.50 µL, 2 mg/mL in 10 mM PB pH 7.4, 150 mM NaCl; molar ratio 2.33:1) following General Procedure II. An EE of 81% was determined for lysozyme-Cy5.

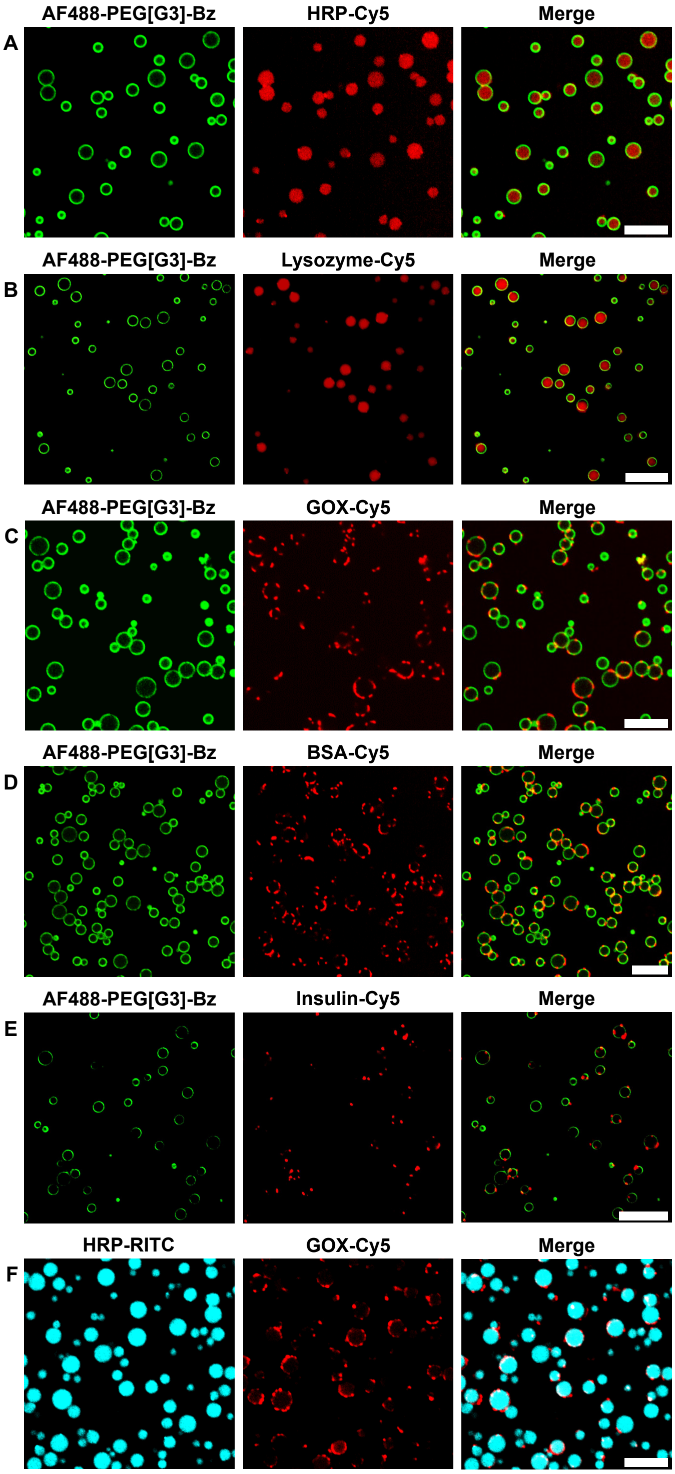

Figure S4. CLSM images of MCM interfacially stabilized with AF488-PEG[G3]-Bz show HRP-Cy5/HRP-RITC (**A**, **F**) and lysozyme-Cy5 (**B**) dispersing throughout the coacervate interior. Conversely, GOX-Cy5 (**C**, **F**), BSA-Cy5 (**D**), and insulin-Cy5 (**E**) form discrete localized patches at the MCM periphery. Scale bars 10 µm, AF488 (green), Cy5 (red), RITC (cyan).

6. Transfer of Macromolecular Components Between Dendritic MCM Populations

PLL-Cy5/MCM and PLL-AF488/MCM. Cy5- and AF488-labelled MCM were prepared as described above from solutions of PLL-Cy5 (200 μL) and PLL-AF488 (200 μL) with 3[G2]-Bz (100 μL). After 40 min of orbital stirring (350 rpm) at 21 °C in an Eppendorf Thermomixer C, PEG[G3]-Bz (9 μL) was added to each suspension. Then, both MCM populations were centrifuged (12000 g, 5 min) and mixed in a 1:1 ratio to a volume of 200 μL in a microscope slide (Cellvis 4-Chamber microwells 35 mm Glass Bottom Dish with 20 mm, #1.5 cover glass). The mixture was analyzed by CLSM for 1 h.

HRP-Cy5@MCM and PLL-AF488/MCM. HRP-Cy5-loaded MCM were prepared as described above from a mixture of HRP/HRP-Cy5 (2.50 μL, 2 mg/mL), PLL (200 μL), and 3[G2]-Bz (100 μL). AF488-labelled MCM were prepared as described from a mixture of PLL-AF488 (200 μL) and 3[G2]-Bz (100 μL). Both coacervates were left under orbital stirring (350 rpm) for 40 min at 21 °C in an Eppendorf Thermomixer C. Then, PEG[G3]-Bz (9 μL) was added to each suspension and the resulting MCM were centrifuged (12000 g, 5 min) and mixed in a 1:1 ratio to a volume of 200 μL in a microscope slide (Cellvis 4-Chamber microwells 35 mm Glass Bottom Dish with 20 mm, #1.5 cover glass). The mixture was analyzed by CLSM for 1 h.

PLL-AF488/MCM and GOX-Cy5@MCM. AF488-labelled MCM were prepared as described from a mixture of PLL-AF488 (200 μL) and 3[G2]-Bz (100 μL). GOX-Cy5-loaded MCM were prepared as described above from a mixture of GOX/GOX-Cy5 (6.30 μL, 2 mg/mL), PLL (200 μL), and 3[G2]-Bz (100 μL). Both coacervates were left under orbital stirring (350 rpm) for 40 min at 21 °C in an Eppendorf Thermomixer C. Then, PEG[G3]-Bz (9 μL) was added to each suspension and the resulting MCM were

centrifuged (12000 g, 5 min) and mixed in a 1:1 ratio to a volume of 200 μL in a microscope slide (Cellvis 4-Chamber microwells 35 mm Glass Bottom Dish with 20 mm, #1.5 cover glass). The mixture was analyzed by CLSM for 20 min.

HRP-RITC@MCM and GOX-Cy5@MCM. HRP-RITC-loaded MCM were prepared as described above from a mixture of HRP/HRP-RITC (2.50 μL, 2 mg/mL), PLL (200 μL), and 3[G2]-Bz (100 μL). GOX-Cy5-loaded MCM were prepared as described above from a mixture of GOX/GOX-Cy5 (6.30 μL, 2 mg/mL), PLL (200 μL), and 3[G2]-Bz (100 μL). Both coacervates were left under orbital stirring (350 rpm) for 40 min at 21 °C in an Eppendorf Thermomixer C. Then, PEG[G3]-Bz (9 μL) was added to each suspension and the resulting MCM were centrifuged (12000 g, 5 min) and mixed in a 1:1 ratio to a volume of 200 μL in a microscope slide (Cellvis 4-Chamber microwells 35 mm Glass Bottom Dish with 20 mm, #1.5 cover glass). The mixture was analyzed by CLSM for 20 min.

7. Internal Dynamics within Dendritic MCM. FRAP Measurements

Independent MCM populations containing PLL-Cy5 (100 μL) or encapsulating HRP-Cy5 (100 μL) were transferred to separate wells of a μ-side 18-well glass-bottom dish (Ibidi). FRAP experiments were performed employing the FRAP interface available in the Leica LAS X software. For image acquisition, samples were excited with a white light laser (WLL2; λ_{ex} 649 nm, λ_{em} 654-700 nm) and visualized with an HC PL APO CS2 93×/1.30 Gly objective. An initial image was acquired to define a region of interest (ROI) of 1.2 μm × 1.2 μm, corresponding to approximately one-fourth of a coacervate droplet. Then, a 3 image series (256 × 125 μm) was acquired before bleaching the ROI using 20 iterations at 649 nm (100% laser power). Fluorescence recovery was monitored for 1.5 min after photobleaching by acquiring a 40 image series at 0.05% laser power.

Fluorescence intensities of the ROI (*S*), a reference area (a nearby coacervate not bleached, *R*), and the background (*B*) were extracted from the images using ImageJ software. Then, recovery data were normalized to background and reference area using Eq S2,[3,4,5] where *F*(*t*) is the normalized fluorescence intensity of the ROI at a given time (*t*).

$$F(t) = \frac{[S(t) - B(t)]\ [R(0) - B(0)]}{[R(t) - B(t)]\ [S(0) - B(0)]} \qquad \text{Eq S2}$$

Afterwards, *F*(t) data were fitted to a first-order exponential equation (Eq S3) using Origin 2022 software (OriginLab)

$$F(t) = A\left(1 - \mathrm{e}^{-\frac{t}{\tau}}\right) + C \qquad \text{Eq S3}$$

where τ is the fluorescence recovery time constant, *A* is the amplitude of the recovery, and *C* the intercept (Figure S5).

The fluorescence recovery half-life ($t_{1/2}$) was determined from Eq S4 and used to calculate the apparent diffusion coefficient (D_{app}) using Eq S5,[5,6] where ω is the radius of the ROI.

$$t_{1/2} = \tau \ln 2 \qquad \text{Eq S4}$$

$$D_{app} = 0.88\ \omega^2/4\ t_{1/2} \qquad \text{Eq S5}$$

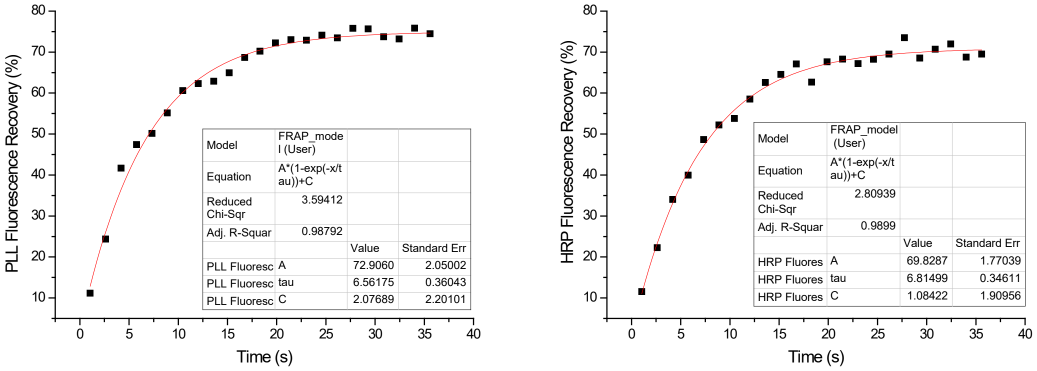

Figure S5. Fitting of the FRAP recovery curves of PLL-Cy5/MCM and HRP-Cy5@MCM to Eq S3.

8. Intra-MCM GOX-HRP Enzymatic Cascade Reaction

Monitoring Intra-MCM GOX-HRP Enzymatic Cascade Reaction by CLSM. A 90 μL sample of GOX-Cy5/HRP-RITC@MCM in 10 mM PB pH 7.4, 150 mM NaCl was deposited onto a microscope slide (Cellvis 4-Chamber microwells 35 mm Glass Bottom Dish with 20 mm, #1.5 cover glass). After the addition of *o*-phenylenediamine (oPD, 5 μL, 10 mM in 10 mM PB pH 7.4, 150 mM NaCl), an image was acquired at t = 0. Then, the enzymatic cascade reaction was triggered by gently pipetting glucose (5 μL, 20 mM in 10 mM PB pH 7.4, 150 mM NaCl) into the coacervate suspension (final enzyme concentrations: 80 nM GOX and 75 nM HRP). The sample was imaged at different time points to monitor the production and localization of the reaction product 2,3-diaminophenazine (2,3-DAP). Control experiments performed under identical conditions in the absence of glucose or using MCM lacking GOX or HRP did not result in the production of 2,3-DAP (Figure S6).

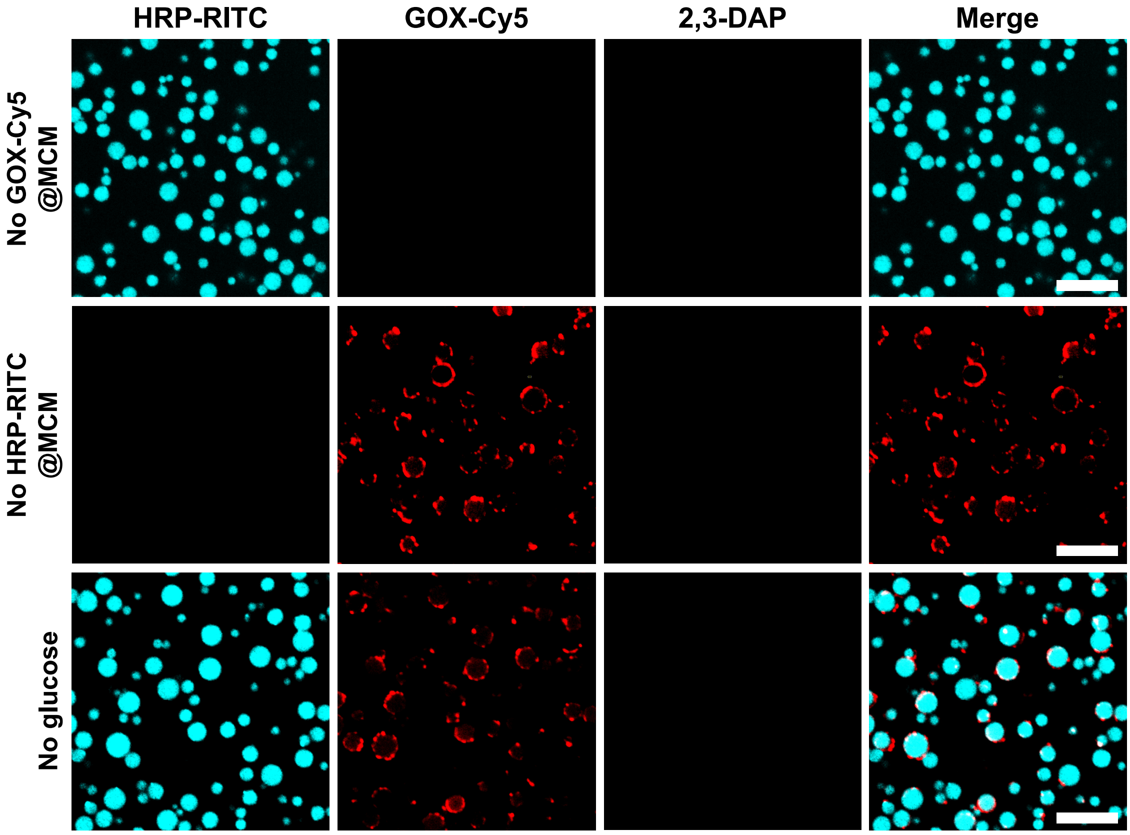

Figure S6. CLSM images (20 min) of control GOX-HRP enzymatic cascade experiments performed with MCM lacking GOX or HRP, or in the absence of glucose. Scale bars 10 μm.

Monitoring Intra-MCM GOX-HRP Enzymatic Cascade Reaction by Absorbance. A 95 µL sample of GOX/HRP@MCM in 10 mM PB pH 7.4, 150 mM NaCl, was deposited onto a 96-well plate (Thermo Fisher Flat Black MultiWells). ABTS (2.50 µL, 3.2 mM in 10 mM PB pH 7.4, 150 mM NaCl) and glucose (2.50 µL, 160 mM in 10 mM PB pH 7.4, 150 mM NaCl) were sequentially added to initiate the enzymatic reaction (final enzyme concentrations: 80 nM GOX and 75 nM HRP). The reaction progress was monitored by measuring the absorbance of the ABTS radical cation (405 nm) in a plate reader (Tecan Infinite F200 pro). Absorbance was corrected for the scattering of the coacervate suspension and normalized. Control experiments performed under identical conditions in assays without glucose or using MCM lacking GOX or HRP resulted in zero increase in absorbance in all cases.

9. Synthetic Cell-Natural Cell Communication Assays: A549

A549 Culture. Human adenocarcinoma alveolar basal epithelial (A549) cells, obtained from the European Collection of Authenticated Cell Cultures (ECACC), were cultured at 37 °C in a 5% CO_2 atmosphere in Dulbecco's modified Eagle's medium (DMEM) with high glucose, containing 10% fetal bovine serum (FBS) and supplemented with 50 U/mL penicillin and 50 U/mL streptomycin, in 75 cm^2 cell culture flasks. All cell experiments were performed at 37 °C in a 5% CO_2 atmosphere with this modified DMEM, referred in the text as "medium".

Cell Viability. MCM prepared as described above at a concentration of 2.05 mg/mL in 10 mM PB pH 7.4, 150 mM NaCl were diluted with medium to reach final concentrations between 250 and 15 μg/mL. A549 cells were seeded in 96-well plates at a density of 100000 cells/mL and incubated at 37 °C in 5% CO_2 for 24 h. Then, the medium was replaced with the MCM solutions (100 μL) – or solutions of the polyelectrolyte constituents 3[G2]-Bz, PEG[G3]-Bz, and PLL at the same concentrations as in MCM – and incubation continued for 48 h. Cell viability was determined by a colorimetric assay with CCK-8 following the manufacturer's protocol. After 2 h of incubation with a 6% solution of CCK-8 in fresh medium, supernatant solution was transferred to a 96-well culture plate (Corning Costar 96-Well microplates from Thermo Scientific). Viability was determined by measuring the supernatant absorbance at 450 nm in a plate reader Tecan Infinite F200 PRO. Absorbance (A) from a 6% CCK-8 solution in medium was subtracted from all data points. Viability was calculated as follows:

$$\text{Cell Viability (\%)} = 100 \times \frac{(A_{\text{sample}} - A_{6\%\ \text{CCK-8}})}{(A_{\text{control}} - A_{6\%\ \text{CCK-8}})} \qquad \text{Eq S6}$$

Monitoring MCM-A549 Cell Communication by CLSM. A549 cells were seeded onto a microscope slide (Cellvis 4-Chamber microwells 35 mm Glass Bottom Dish with 20 mm, 1.5 cover glass) at a density of 150000 cells/mL. After 24 h of incubation at 37 °C in 5% CO_2, the culture medium was replaced with 200 μL of fresh medium and 45 μL of GOX-Cy5/HRP-RITC@MCM. Then, oPD (5 μL, 26 mM in 10 mM PB pH 7.4, 150 mM NaCl) was added to initiate the enzymatic cascade reaction (80 nM GOX and 75 nM HRP). The sample was imaged at different time points to monitor the production and localization of the reaction product 2,3-DAP. Control experiments performed under identical conditions in the absence of any of the enzymes or oPD did not result in the production of 2,3-DAP (Figure S7).

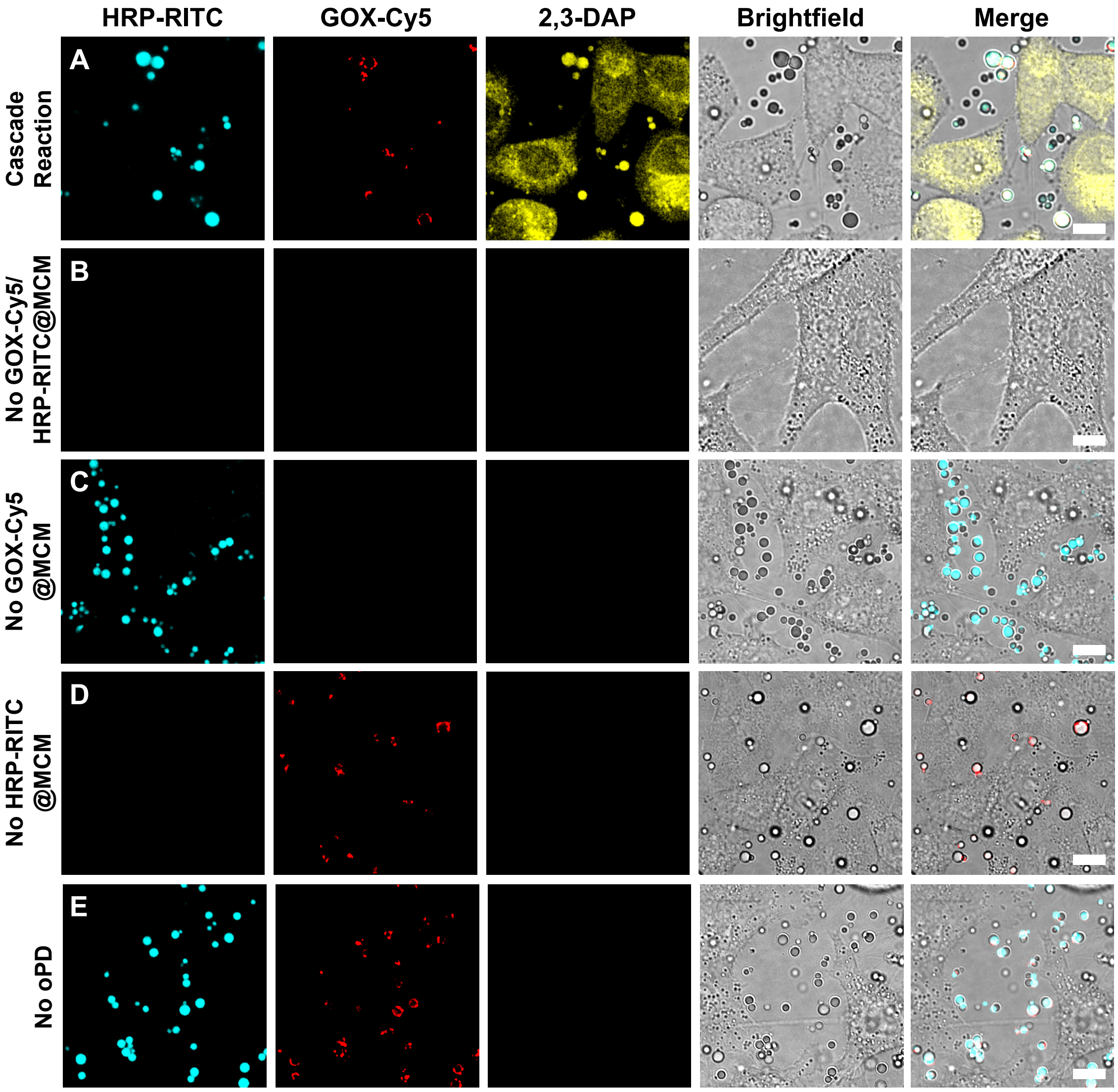

Figure S7. CLSM and brightfield microscopy images (20 min) of the enzymatic production of 2,3-DAP (yellow) – from GOX-Cy5/HRP-RITC@MCM (red and cyan, respectively), oPD, and glucose (present in the culture medium) – and its migration to A549 cells (**A**). Control experiments (20 min) performed in the absence of GOX-Cy5/HRP-RITC@MCM (**B**), with MCM populations lacking GOX-Cy5 (**C**) or HRP-RITC (**D**), or without oPD (**E**) show no production of 2,3-DAP. Scale bars 10 µm.

10. Synthetic Cell-Natural Cell Communication Assays: Red Blood Cells (RBC)

Isolation of RBC. Fresh rat blood was collected and immediately mixed with sodium citrate to a final concentration of 25 mM. RBC were obtained after multiple rounds of centrifugation in cold PBS, until the supernatant became clear (1600 *g* at 4 °C for 10 min). A 3% (w/v) suspension of RBC was then prepared in PBS.

Monitoring Oxidation of Oxyhemoglobin (oxyHb) to Methemoglobin (metHb) by UV-Vis Spectroscopy. A sample of RBC (150 µL, 0.1% w/v in PBS) was deposited onto a 96-well plate (Thermo Fisher Flat Black MultiWells). GOX@MCM (30 µL) and glucose (15 µL, 1 M in 10 mM PB pH 7.4, 150 mM NaCl) were sequentially added to initiate the reaction (46 nM GOX). Oxidation progress was monitored by UV-Vis spectroscopy (325 -750 nm) using a plate reader (Tecan Infinite F200 pro) at 5 min intervals. Before each measurement, the plate was shacked for 1 s to homogenize the medium. Absorbance was corrected at 750 nm for the scattering of the coacervate suspension and normalized. Oxidation of oxyHb to metHb was confirmed by a shift of the Soret band from 414 to 404 nm, the disappearance of oxyHb absorbance peaks at 540 and 578 nm, and the emergence of characteristic metHb signals at 500 and 630 nm.[7]

Monitoring MCM-RBC Communication with oPD by CLSM. Aliquots of RBC suspension (150 µL, 1% w/v in PBS) were deposited onto a 24-well plate (Cellvis, 15 mm glass bottom), followed by GOX-Cy5@MCM (30 µL). Then, oPD (30 µL, 3 mM in 10 mM PB pH 7.4, 150 mM NaCl) was added and an image was acquired at t = 0. Then, the enzymatic cascade reaction was triggered by gently pipetting glucose (15 µL, 1 M in 10 mM PB pH 7.4, 150 mM NaCl) into the suspension (final concentration 40 nM GOX). The sample was imaged at different time points to monitor the production and localization of the reaction product 2,3-DAP. Control experiments carried out using a blank MCM

(no GOX) or in the absence of GOX-Cy5@MCM or glucose did not produce any fluorescence redout (Figure S8).

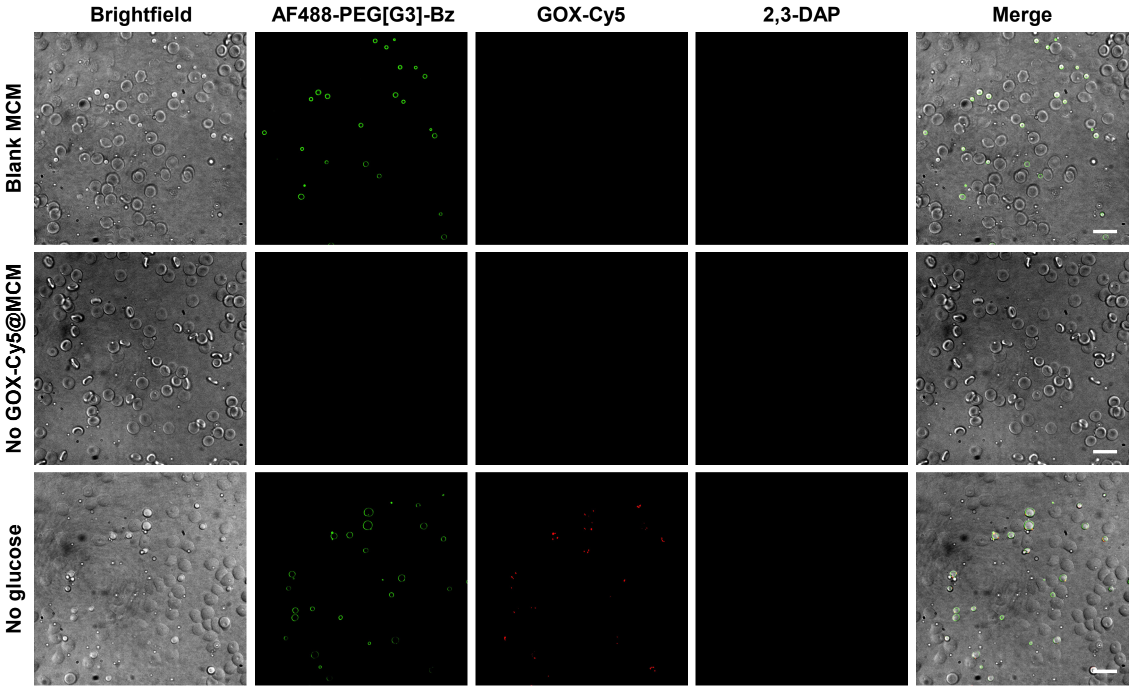

Figure S8. CLSM and brightfield microscopy images (10 min) of control MCM-RBC communication experiments performed with oPD using a blank MCM (no GOX) or in the absence of GOX-Cy5@MCM or glucose. Scale bars 10 µm.

Monitoring MCM-RBC Communication with oPD by Fluorescence Spectroscopy. Aliquots of RBC suspension (150 μL, 1% w/v in PBS) were deposited onto a 24-well plate (Cellvis, 15 mm glass bottom), followed by GOX@MCM (30 μL). Then, oPD (30 μL, 3 mM in 10 mM PB pH 7.4, 150 mM NaCl) and glucose (15 μL, 1 M in 10 mM PB pH 7.4, 150 mM NaCl) were sequentially added to initiate the enzymatic cascade (final concentration 40 nM GOX). The reaction progress was monitored by measuring the fluorescence of the reaction product 2,3-DAP (λ_{ex} 405 nm, λ_{em} 550 nm) in a plate reader (Tecan Infinite F200 PRO). Control experiments showed no fluorescence when the assay was performed using a blank MCM (no GOX) or in the absence of GOX@MCM or glucose (Figure S9).

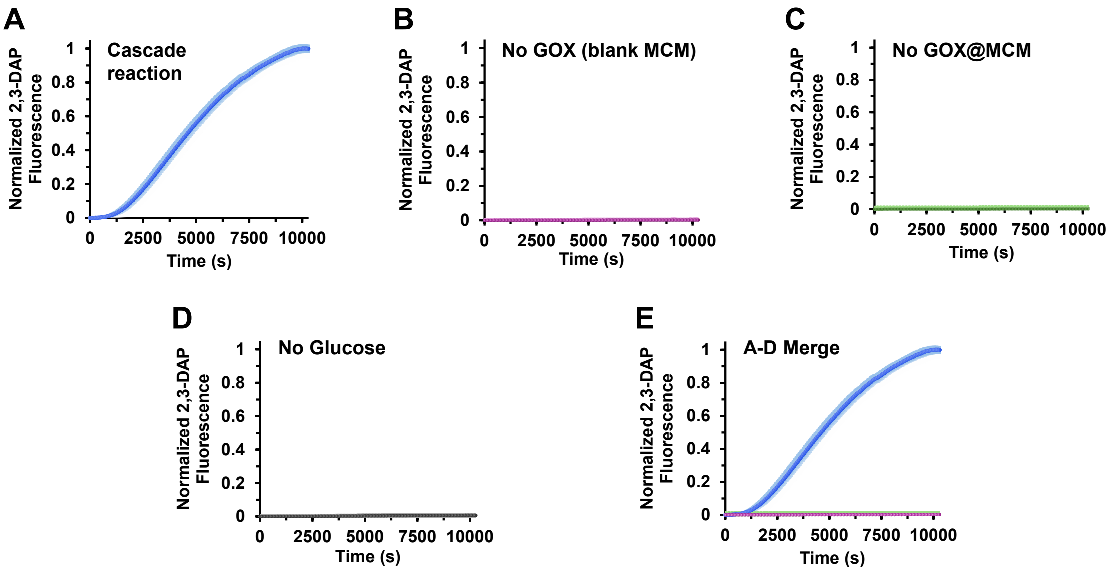

Figure S9. Progress of the enzymatic cascade between GOX@MCM and RBC (glucose and oPD as substrates) studied by monitoring the fluorescence of the reaction product 2,3-DAP (**A**). Control experiments performed using a blank MCM (no GOX, **B**), or in the absence of GOX@MCM (**C**) or glucose (**D**). Merge of A-D (**E**).

Monitoring MCM-RBC Communication with Amplex Red by CLSM. Aliquots of RBC suspension (150 µL, 1% w/v in PBS) were deposited onto a 24-well plate (Cellvis, 15 mm glass bottom), followed by Amplex Red (5 µL, 80 µM in DMSO). The suspension was incubated at rt for 45 min, after which the presence of Amplex Red inside RBC was confirmed by CLSM via photooxidation to resorufin (λ_{ex} 561 nm, λ_{em} 620/50 nm). Subsequently, the excitation and acquisition settings were adjusted to ensure that no resorufin fluorescence was detected. Then, GOX-Cy5@MCM (30 µL) was added and an image was acquired at t = 0 (Figure S10). The enzymatic cascade reaction was triggered by gently pipetting glucose (15 µL, 1 M in 10 mM PB pH 7.4, 150 mM NaCl) into the suspension (final concentration 45 nM GOX). The sample was imaged at different time points to monitor the production and localization of the reaction product resorufin. Control experiments carried out using a blank MCM (no GOX) or in the absence of GOX-Cy5@MCM or glucose did not produce any fluorescence redout (Figure S11).

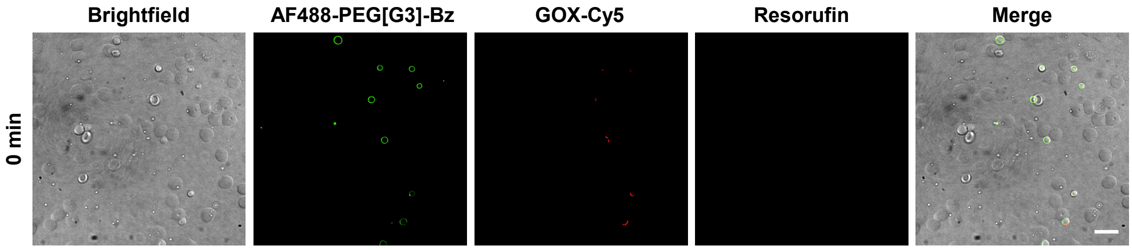

Figure S10. CLSM and brightfield microscopy images of the MCM-RBC communication experiment performed with Amplex Red before the addition of glucose. Scale bar 10 µm.

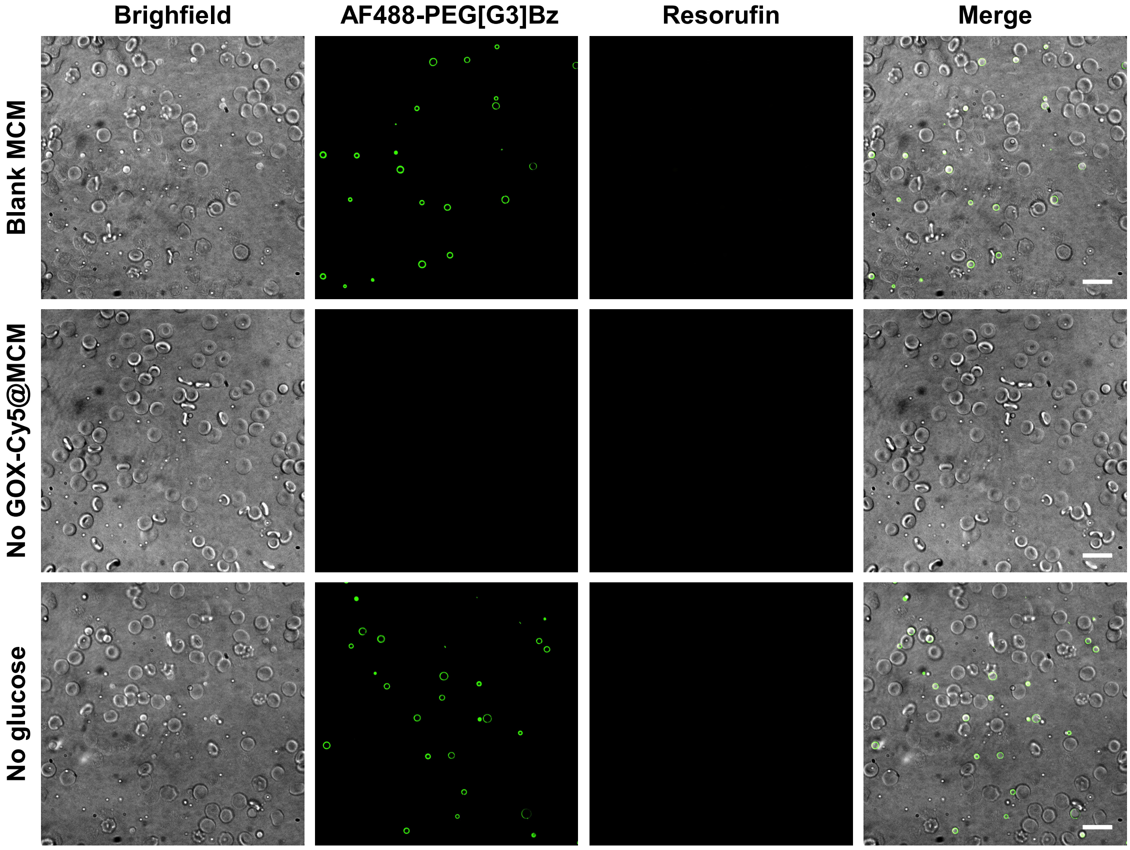

Figure S11. CLSM and brightfield microscopy images (15 min) of control MCM-RBC communication experiments performed with Amplex Red using a blank MCM (no GOX) or in the absence of GOX-Cy5@MCM or glucose. Scale bars 10 µm.

11. References

1. Fernandez-Villamarin, M.; Sousa-Herves, A.; Porto, S.; Guldris, N.; Martinez-Costas, J.; Riguera, R.; Fernandez-Megia, E. A Dendrimer-Hydrophobic Interaction Synergy Improves the Stability of Polyion Complex Micelles. *Polym. Chem.* **2017**, *8*, 2528-2537.

2. Lopez-Blanco, R.; Magana Rodriguez, J. R.; Esquena, J.; Fernandez-Megia, E. From nanometric to giant polyion complex micelles via a hierarchical assembly of dendrimers. *J. Colloid Interf. Sci.* **2025**, *687*, 293-302.

3. Phair, R. D.; Gorski, S. A.; Misteli, T. In *Methods Enzymol.*; Academic Press: 2003; Vol. 375, p 393-414.

4. Jia, T. Z.; Hentrich, C.; Szostak, J. W. Rapid RNA Exchange in Aqueous Two-Phase System and Coacervate Droplets. *Orig. Life Evol. Biosph.* **2014**, *44*, 1-12.

5. Aumiller, W. M.; Pir Cakmak, F.; Davis, B. W.; Keating, C. D. RNA-Based Coacervates as a Model for Membraneless Organelles: Formation, Properties, and Interfacial Liposome Assembly. *Langmuir* **2016**, *32*, 10042-10053.

6. Axelrod, D.; Koppel, D. E.; Schlessinger, J.; Elson, E.; Webb, W. W. Mobility measurement by analysis of fluorescence photobleaching recovery kinetics. *Biophys J.* **1976**, *16*, 1055-1069.

7. Ledvina, M. Rapid spectrophotometric determination of carbonylhemoglobin in blood. *Biochem. Clin. Bohemoslov.* **1987**, *16*, 493-495.
